# Transcription factor binding specificities of the oomycete *Phytophthora infestans* reflect conserved and divergent evolutionary patterns and predict function

**DOI:** 10.1186/s12864-024-10630-6

**Published:** 2024-07-23

**Authors:** Nguyen N. T. Vo, Ally Yang, Wiphawee Leesutthiphonchai, Yulong Liu, Timothy R. Hughes, Howard S. Judelson

**Affiliations:** 1 Department of Microbiology and Plant Pathology, University of California, Riverside, CA 92521 USA; 2 Department of Molecular Genetics and Donnelly Center, University of Toronto, Toronto, ON M5S 3E1 Canada; 3 Current address: Department of Plant Pathology, Faculty of Agriculture, Kasetsart University, Bangkok, 10900 Thailand

**Keywords:** Transcription factor binding site, Protein-binding oligonucleotide microarray, Oomycete, Promoter, Phytophthora infestans, Gene regulation, DNA-binding protein

## Abstract

**Background:**

Identifying the DNA-binding specificities of transcription factors (TF) is central to understanding gene networks that regulate growth and development. Such knowledge is lacking in oomycetes, a microbial eukaryotic lineage within the stramenopile group. Oomycetes include many important plant and animal pathogens such as the potato and tomato blight agent *Phytophthora infestans*, which is a tractable model for studying life-stage differentiation within the group.

**Results:**

Mining of the *P. infestans* genome identified 197 genes encoding proteins belonging to 22 TF families. Their chromosomal distribution was consistent with family expansions through unequal crossing-over, which were likely ancient since each family had similar sizes in most oomycetes. Most TFs exhibited dynamic changes in RNA levels through the *P. infestans* life cycle. The DNA-binding preferences of 123 proteins were assayed using protein-binding oligonucleotide microarrays, which succeeded with 73 proteins from 14 families. Binding sites predicted for representatives of the families were validated by electrophoretic mobility shift or chromatin immunoprecipitation assays. Consistent with the substantial evolutionary distance of oomycetes from traditional model organisms, only a subset of the DNA-binding preferences resembled those of human or plant orthologs. Phylogenetic analyses of the TF families within *P. infestans* often discriminated clades with canonical and novel DNA targets. Paralogs with similar binding preferences frequently had distinct patterns of expression suggestive of functional divergence. TFs were predicted to either drive life stage-specific expression or serve as general activators based on the representation of their binding sites within total or developmentally-regulated promoters. This projection was confirmed for one TF using synthetic and mutated promoters fused to reporter genes *in vivo.*

**Conclusions:**

We established a large dataset of binding specificities for *P. infestans* TFs, representing the first in the stramenopile group. This resource provides a basis for understanding transcriptional regulation by linking TFs with their targets, which should help delineate the molecular components of processes such as sporulation and host infection. Our work also yielded insight into TF evolution during the eukaryotic radiation, revealing both functional conservation as well as diversification across kingdoms.

**Supplementary Information:**

The online version contains supplementary material available at 10.1186/s12864-024-10630-6.

## Background

Transcription factors (TFs) establish patterns of gene expression by binding specific sequences in DNA which are usually 5’ of the target gene and 6 to 12 nt in size [1]. Over 70 families of eukaryotic TFs have been identified and classified based on the structure of their DNA-binding domains. Some families are restricted to specific taxonomic groups while others occur across kingdoms [2, 3]. Changes in TF binding specificities underlie important evolutionary processes [4, 5] since many of the proteins are master regulators, capable of activating or repressing expression in a tissue or condition-specific manner. Other TFs serve as general activators or direct RNA polymerase to a specific start site. Many TFs act in concert with cofactor proteins or other partners [6]. For example, Basic Leucine Zipper Domain TFs (bZIP) TFs usually act as homo- or heterodimers, while canonical Heat Shock Factors (HSFs) form homo- or heterotrimers [7, 8]. The binding preferences of TFs are typically described as motifs containing a mix of invariable and degenerate sites [9]. Identifying these motifs is an important step towards understanding the function of a TF.

Studies of TFs are relatively limited in the filamentous microbe *Phytophthora infestans*, an oomycete member of the stramenopile lineage of eukaryotes [10]. A few TFs have been shown to regulate its life cycle or those of close relatives [11–14]. *P. infestans* is a devastating pathogen of potato and tomato and is notorious as a cause of the Irish Famine of the 1840’s [15]. It is a useful model for oomycetes since it can be cultured on artificial media or plants, technologies exist for manipulating genes [16–18], and a chromosome-scale genome assembly is available [19]. The asexual life cycle of *P. infestans* involves the growth of branched vegetative hyphae which extract nutrients from media or a plant host [20]. As cultures or plant lesions age, the hyphae produce multinucleate sporangia capable of traveling in wind or water to new hosts. Upon chilling in a moist environment, the cytoplasm of each sporangium cleaves into individual zoospores which swim, encyst, and produce germ tubes able to penetrate host tissues. There is also a sexual cycle that occurs when the hyphae of opposite mating types (A1 and A2) interact, causing gametangia to differentiate which unite to generate oospores [21].

Changes in gene expression during the *P. infestans* life cycle are extensive. RNA-seq analyses have shown that of the approximately 17,000 genes expressed from the 219 Mb genome, as many as 49% show more than a 5-fold change in mRNA abundance between life-stages with 8% showing greater than a 100-fold change [22–24]. Examples of important genes that vary during these transitions include those encoding regulators of mitotic dormancy in spores [25], structural components of zoospore flagella [26], or effectors that suppress host defenses during early plant infection or cause host cell death during late infection [27]. Despite the obvious importance of TFs in such processes, the DNA target of only one oomycete TF has been identified [13]. Being able to describe the DNA-binding specificities of *P. infestans* TFs will help illuminate the transcriptional networks of these important but understudied microbes.

Functional binding sites for most TFs in *P. infestans* are thought to reside close to the gene since intergenic distances average only 430 nt and 5’ untranslated regions are typically smaller than 50 nt [28, 29]. Distant enhancers seem unlikely to play a major role in transcriptional control since adjacent genes usually have distinct patterns of expression [28]. Also, studies with reporter genes have shown that about 250 nt of DNA upstream of the transcription start site is sufficient to drive normal expression [18, 30]. Consequently, bioinformatic approaches for identifying TF binding sites have generally focused on the 500 nt upstream of the translation start site. Several motifs have been predicted bioinformatically but remain unlinked to a specific TF [28, 31, 32].

In the present study, we used protein binding microarrays (PBMs; [33, 34]) to successfully define the DNA binding preferences of 73 sequence-specific TFs representing the major families in *P. infestans.* Several targets were confirmed using electrophoretic mobility shift assays (EMSA) or chromatin immunoprecipitation (ChIP). We observed that paralogs bearing similar DNA-binding domains often bound related targets and clustered in the genome but displayed distinct patterns of expression consistent with neo- and subfunctionalization models of gene duplication [35]. Motif enrichment analysis combined with studies using a reporter gene suggested which TFs may serve as general activators or stage-specific regulators. About half of the *P. infestans* TFs bound sequences resembling the targets of related proteins from human and plants, reflecting both functional conservation as well as diversification across kingdoms.

## Methods

### Identification of TFs and tree construction

Two parallel methods were used to identify TFs from *P. infestans*, using gene models from strain T30-4 [36]. One approach, implemented in the Cis-BP pipeline (http://cisbp.ccbr.utoronto.ca), relied mostly on PFAM domains [37]. The second method searched for INTERPRO domains [38]. The two lists were compared and manually curated. As part of this process, predicted genes lacking expression in 16 developmental stages and growth conditions based on RNA-seq data [22–24, 39] were considered to be pseudogenes and removed from the list of TF candidates. Also eliminated were several genes resulting from apparent false duplications in the T30-4 assembly.

For species other than *P. infestans*, domain searches were performed using genome data in Fungidb or Ensembl Protists (http://protists.ensembl.org) or Fungidb [40]. For *P. infestans* and several other species, putative interaction domains not defined by PFAM or INTERPRO were identified using Waggawagga and Deepcoil [41, 42]. Phylogenetic trees were constructed from the DNA-binding domains using MUSCLE and PhyML as implemented in SEAVIEW [43] with 1000 bootstrap replicates.

### RNA-seq

Expression analyses were performed using tissues and fastq files from prior studies using an average of three biological replicates [22–24, 39]. In brief, RNA was isolated using kits from Sigma or Agilent. RNA-seq was performed using indexed libraries prepared using the Illumina Truseq kit, and sequenced to produce 75-nt single-end reads. Reads passing the quality filter were aligned to the *P. infestans* T30-4 genome using Bowtie 2.2.5 and Tophat version 2.0.14, allowing for one mismatch [44]. Expression and differential expression calls were made with edgeR [45]. Data for the heatmaps were from isolate 1306 except that the mating data reflects the average of three crosses: 88069 × 618, 8811×E13, and 88069×E13. Heatmaps were generated using Seaborn [46] using per-gene normalized data. The latter were obtained by dividing FPKM values for each gene against the mean of that gene across all conditions, such that the mean value across the heatmap for that gene would equal 1.0. Thus, the reads in each gene are scaled similarly which allows gene expression across conditions in the heatmap to be compared.

### Protein Binding Microarrays (PBMs)

For each TF, parallel arrays were analyzed using recombinant protein produced in *E. coli* and by in vitro transcription-translation. These assays included the PFAM-defined DNA-binding domain, 50 flanking N-terminal and 50 flanking C-terminal residues (or until the end of the protein), and a 6×His tag. The protein sequences used are shown in Table S1. These included oligomerization domains such as leucine zippers and other coiled-coil domains since they were either included in the PFAM domain (e.g., bZIP and HLH TFs) or resided within the 50 amino acids adjacent to that domain (e.g., HSF and HTH TFs). After optimization for expression in *E. coli*, the sequences were synthesized and cloned into pTH6838 or pTH7069, which contain a T7 promoter and add an N or C-terminal glutathione S-transferase (GST) tag, respectively [33]. In vitro transcription-translation was performed using the PURExpress In Vitro Protein Synthesis Kit from New England BioLabs. For proteins produced in *E. coli*, soluble proteins were purified using nickel resin and eluted in phosphate-buffered saline (PBS) containing 10 to 20% glycerol. Proteins in inclusion bodies were first solubilized in 2 M urea in PBS and then refolding was achieved in 0.5 M arginine containing 10 to 20% glycerol, which was adjusted through dialysis to phosphate-buffered saline with 10% glycerol. Gel analysis indicated that the proteins were 85 to 90% pure and of the expected size.

The methods for analyzing the arrays were as described [33, 34, 47]. Each TF was analyzed in duplicate on two different arrays with differing probe sequences (HK and ME), from which positive 8-mers were identified and E- and Z-scores calculated as described [48]. Experiments were judged as successful if at least one 8-mer had an E-score above 0.45 on both arrays, if the two arrays yielded correlated E- and Z-scores, and if the arrays defined similar motifs based on alignments of the 8-mers using Top10AlignZ [37].

### Electrophoretic mobility shift assays

These were performed as described [49] using ca. 35-nt double-stranded DNAs and a Cy5 label. The oligonucleotide sequences, DNA concentrations, and protein concentrations employed are shown in Table S2. Preliminary titrations established conditions where DNA was in excess. After incubation for 15 min at room temperature, the mixtures were separated on a 5% acrylamide non-denaturing gel and imaged with a Typhoon laser scanner. Dissociation constants were calculated based on band intensities measured with ImageJ [50].

### Reporter gene analysis

Vectors for *P. infestans* transformation were based in either the promoter-less reporter plasmid pNPGUS or a derivative containing the 74-nt minimal *NifS* promoter [28]. The sequences shown in Table S3 were generated by polymerase chain reaction or synthesized and then cloned upstream of the GUS reporter. Transformants of strain 1306 were obtained by the protoplast method using G418 selection [51]. Transformant tissue was disrupted by grinding in liquid nitrogen and assayed using bromochloroindoyl-β-glucuronide or 4-methylumbelliferyl glucuronide [31].

### Binding site enrichment analysis

Lists of genes upregulated in a developmental or infection stage were identified using RNA-seq data [22–24, 39]. This involved comparisons to nonsporulating mycelia grown on rye-sucrose, except for nonsporulating mycelia and mating cultures which were compared to sporangia and single cultures of the parents, respectively. For each gene list as well as control lists (i.e., the remaining genes), 500-nt putative promoters were extracted and scanned for the motifs using FIMO using a *p*-value cut-off of 10^− 4^ [52]. The statistical significance of over- or under-representation was measured by Chi-Square tests.

### Chromatin immunoprecipitation

Triplicate samples of sporangia and sporulating hyphae were collected in 10 mM Tris pH 8.0, 10 mM MgCl_2_, 0.4 M sucrose, and 1 mM phenylmethylsulfonyl fluoride (PMSF). After adding 1.5 or 2% formaldehyde, the mixture was shaken for 15 min at 50 rpm and glycine was added to 0.125 M. After 5 min of further shaking, the tissue was pelleted at 700×*g* for 4 min, washed three times in PBS pH 7.4, frozen in liquid nitrogen, and ground using a mortar and pestle. After resuspension in buffer A (10 mM Tris pH 8.0, 0.25 M sucrose, 10 mM MgCl_2_, 1% Triton X-100, 1% protease inhibitor cocktail, 1% PMSF), the material was vortexed for 30 s, subjected to 20 strokes in a tight-fitting Dounce homogenizer, incubated on ice for 1 h, and passed through 15 μm mesh. Nuclei were pelleted from the flow-through at 1,800×*g* at 4ºC for 10 min. After shearing the chromatin to 100–300 nt in a Covaris S220 sonicator, samples were incubated overnight with gentle rocking at 4ºC with mouse IgG or a custom MADS antibody. Protein A magnetic beads (Surebeads, Bio-Rad) were then added and gently mixed for 3 h at 4ºC. Using a magnetic stand, the beads were then washed twice with Buffer A lacking protease inhibitors, twice with Buffer B (100 mM Tris pH 8.0, 500 mM LiCl, 1% Triton X-100, 1% deoxycholic acid), and once with Buffer C (Buffer B plus 150 mM NaCl). The beads were eluted by shaking at 800 rpm for 30 min at room temperature with 0.1 ml of Buffer D (100 mM NaHCO_3_, 1% SDS). A small portion (5 µl) was saved for immunoblot analysis to confirm the presence of MADS protein, with the rest (95 µl) used for DNA extraction. The latter entailed adding NaCl to 0.54 M followed by overnight incubation at 65ºC to reverse formaldehyde crosslinks. Then, 2 µl of 20 mg/ml Proteinase K was added and incubated for 2 h at 45ºC. The material was mixed with an equal volume of 25:24:1 phenol: chloroform: isoamyl alcohol for 5 min followed by separation at 10,000×*g* for 10 min, and then mixed for 2 min with 24:1 chloroform: isoamyl alcohol followed by 2 min of centrifugation at 10,000×*g*. To the aqueous phase was added sodium acetate pH 5.2 to 0.3 M, glycogen to 1 µg/µl, and 2.5 volumes of cold ethanol. After overnight incubation at -20ºC, the DNA was pelleted at 15,000×*g* for 20 min, washed with 95% ethanol, dried for 10 min, and dissolved in 10 µl of 10 mM Tris pH 8.0. The DNA was then subjected to paired-end Illumina sequencing. Each sample yielded an average of 4.7 million 75-nt reads, which were trimmed using Cutadapt and mapped to the reference genome using Bowtie2 [53]. Peaks were detected using Homer [54]. Motifs enriched in peaks unique to the anti-MADS samples were identified using STREME [55].

## Results

### Genome-wide identification of TFs in *P. infestans* and relatives

**Fig. 1:**
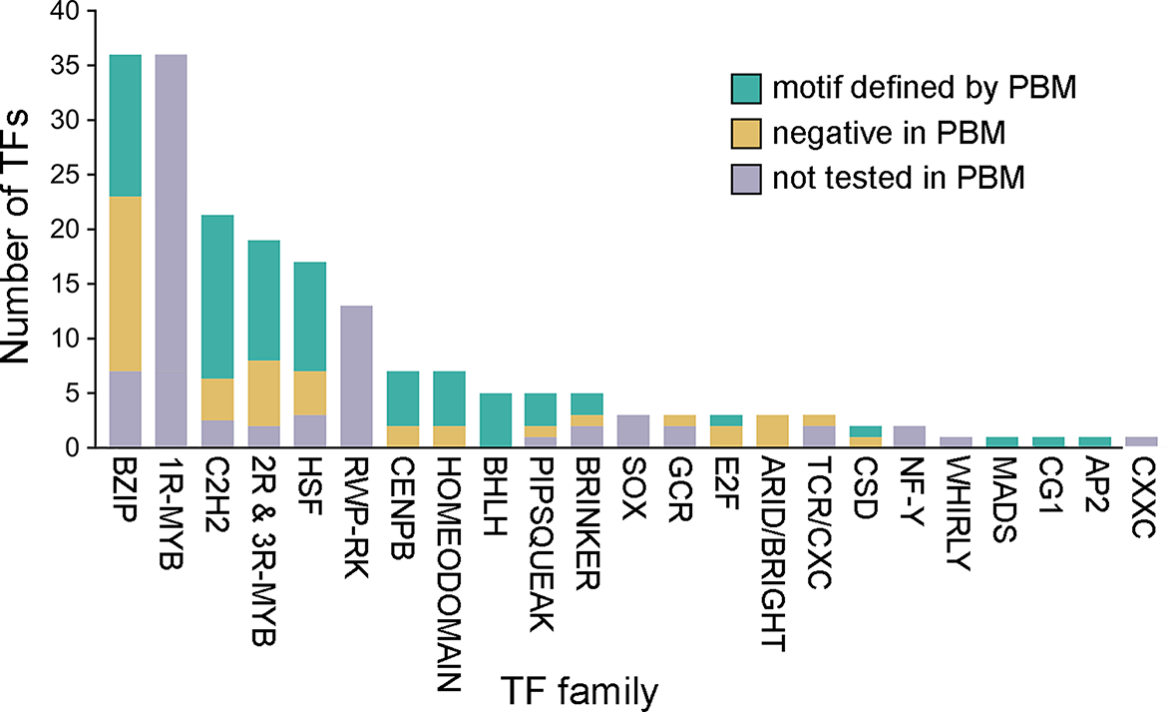
Predicted sequence-specific transcription factors (TFs) from *P. infestans*. Each bar is colored to indicate the number that yielded a DNA-binding motif in the PBMs, as described in Methods. Myb proteins with single DNA-binding domains (1R-Myb) or two two or three such domains (2R and 3R-Mybs) as listed separately since many 1R-Mybs may be telomere-binding factors rather than TFs [56]. Predicted pseudogenes are not included

Of 325 *P. infestans* genes annotated as encoding proteins with DNA-binding activity (GO:0003700), 197 were selected for further study since they belonged to TF families known to exhibit sequence-specific DNA binding. This was trimmed to 190 by eliminating genes (including potential pseudogenes) that lacked expression based on RNA-seq data from nonsporulating mycelia from rye-sucrose and minimal media, sporangia, sporangia undergoing zoosporogenesis, motile zoospores, germinated zoospore cysts, 10-day mating cultures, and early and late stages of potato and tomato infection [22–24, 39]. The 190 genes belonged to 22 families with the largest encoding bZIP, Myb, Heat Shock Factor (HSF), and C2H2 (Cys_2_His_2_) zinc finger proteins (Fig. 1). The figure also indicates the number of proteins in each family that were tested in the PBMs, as will be described in more detail in later sections. The similarity of proteins within each family can be surmised from the phylogenetic trees in Fig. S1.

The chromosomal distribution of the genes was consistent with growth of many of the families through unequal crossing-over. For example, many genes encoding bZIP and C2H2 proteins were clustered (Fig. S2). Such expansions appeared to have been ancient since similar numbers of TFs were detected in most oomycetes including other members of *Phytophthora* and representatives of *Globisporangium*,* Pythium*, and *Saprolegnia* (Table S4). However, none of the enlargements were as extensive as those described for certain families in plants and animals [3]. Some families were smaller in *Hyaloperonospora arabidopsidis* and *Albugo laibachii*, which are obligately pathogenic species having streamlined genomes [57]. Several small families such as GCR1 were not detected in oomycetes besides *Phytophthora.* Most TF families had similar numbers in other stramenopiles, except for the C2H2 group which was about one-fifth the size in diatoms.

### Determination of binding specificities

Protein binding microarrays (PBMs) were used to assay 123 of the *P. infestans* TFs for their DNA-binding specificities. This was limited to the 19 families that had yielded positive results in PBM studies of other species [37, 58]. In brief, this involved expressing their DNA-binding domains and adjacent dimerization domains both in *E. coli* and by coupled in vitro transcription-translation, with GST tags. After incubating the protein fragments with PBMs, fluorophore-conjugated GST antibodies identified the bound oligonucleotides from which TF binding sequences were extracted.

After filtering out low-quality data, binding motifs were generated for 73 TFs by aligning top-scoring 8-mers extracted from the PBMs (Fig. S3). Position-specific frequency matrices (PFMs) based on those alignments are supplied in Fig. S4. These PFMs are also represented by sequence logos that will be presented in the following sections, and the number of proteins within each TF family that successfully yielded a PFM are summarized in Fig. 1. Thirteen of the motifs resembled those associated with *P. infestans* promoters in a prior study [31].

### Heat Shock Factor (HSF) family

**Fig. 2:**
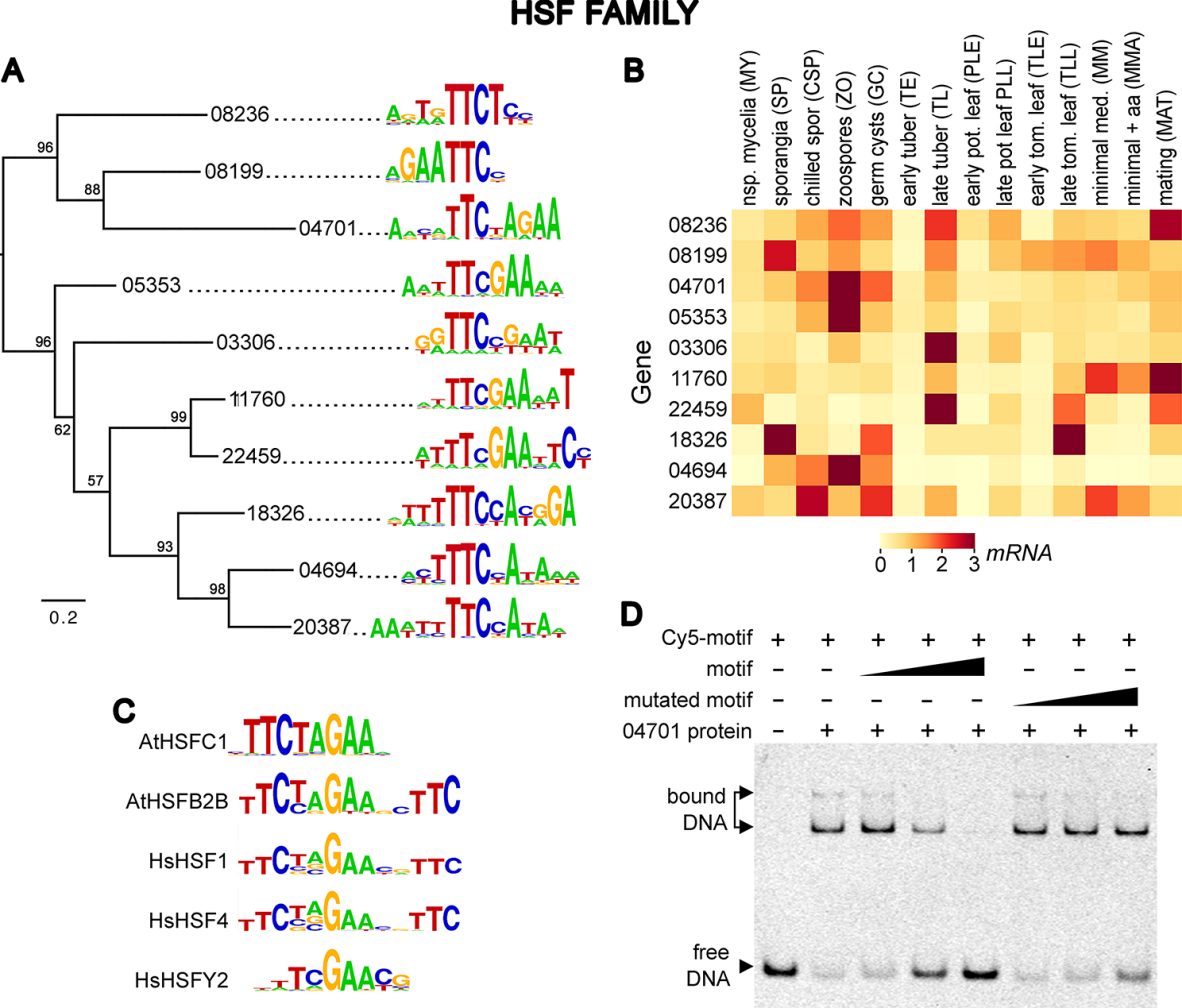
DNA-binding preferences of HSF family TFs. (**A**) Phylogenetic tree based on amino acid sequences of DNA-binding domains, aligned with logo plots representing the predicted DNA targets of each TF. Bootstrap values above 50% are shown at nodes. (**B**) mRNA levels of the TFs in different stages of development including nonsporulating mycelia on rye-sucrose or minimal media, sporangia, sporangia chilled to initiate zoosporogenesis, swimming zoospores, germinating zoospore cysts, infected potato and tomato leaves or potato tuber, and mating cultures. The latter were obtained from 10-day mixed cultures of A1 and A2 hyphae, containing young gametangia and oospores. Expression levels in the heat map are shown as per-gene normalized FPKM values such that the mean of each gene across all samples is 1.0, as described in Methods. A subset of the data was reported previously [22]. (**C**) Binding specificity of HSF TFs from *A. thaliana* (At prefix) or human (Hs prefix) as determined from prior SELEX, DAP-seq, or PBM studies [62, 65–67]. In this and later figures, the human and plant motifs represent those with binding specificities closest to those of the *P. infestans* proteins. Otherwise, representative human and/or plant proteins are shown. (**D**) Validation of PITG_04701 binding site by EMSA using a Cy5-labeled double-stranded oligonucleotide containing TTCTAGAA. The large triangle represents competitors added at 1, 10, and 100-fold the concentration of labeled probe. The results demonstrate specific binding since the same sequence added as an unlabeled competitor reduced the intensity of the retarded bands, unlike DNA containing a mutated motif. A full-length gel is presented in Figure S5

DNA-binding specificities were determined for 10 of the 17 expressed HSFs (Fig. 2A). To help interpret their evolution, in the figure their DNA targets are overlaid on a phylogenetic tree based on the DNA-binding domains. Also shown are expression patterns of the TFs, presented in the same order as in the tree (Fig. 2B). Motifs bound by selected HSFs from human and *Arabidopsis thaliana* are displayed to assess the conservation of the sites across kingdoms (Fig. 2C).

Nearly all characterized HSFs from other eukaryotes including human HSF1 and *A. thaliana* HSFC1 bind sites that contain one or more units of nTTCn [59, 60]. These usually occur in a head-to-head orientation, forming repeats of TTC and GAA separated by a 2-nt gap although HSFs binding head-to-tail arrays are also described [61]. Only a few HSFs, such as human HSFY2, are reported to bind ungapped arrays [62]. Both forms of binding preferences were observed in *P. infestans*. Most common were HSFs that bound ungapped motifs, such as PITG_08199 and PITG_11760 which recognized GAATTC (Fig. 2A). In contrast, the predicted site for PITG_04701 was the gapped motif TTCTAGAA. This target was confirmed by fluorescent EMSA (Fig. 2D).

Whether the *P. infestans* HSFs bound gapped or ungapped sites was incongruent with relationships in the tree (Fig. 2A). For example, the upper-most clade includes HSFs which bound ungapped and gapped dimers, as does the clade in the middle of the tree (e.g., TTCNGAA for PITG_03306 and TTCGAATTC for PITG_22459).

The logos for the three proteins at the base of the tree displayed the TTC motif but with complex flanking positions. Examination of the 8-mers from their PBMs suggested that this was due to flexibility in binding. For example, for both PITG_04694 and PITG_20387 the 8-mers included both ungapped and 2-nt gapped arrays (e.g., TTCGAA and TTCnnGAA) and solo TTC motifs (Fig. S3). Some yeast HSFs have also been shown to bind targets with varying gaps [63].

Adding the human and *A. thaliana* HSFs to phylograms based on the *P. infestans* DNA-binding domains did not support a relationship between binding preference and domain sequence (Fig. S1). In particular, *P. infestans* HSFs that bound gapped or ungapped arrays both clustered with HSFs having gapped targets such as HsHSF1 and AtHSFC1. HsHSFY2, which binds an ungapped target, appeared as an outgroup consistent with a separate evolutionary history. As noted previously, its DNA-binding domain diverges substantially from those of other HSFs [64].

**Fig. 3:**
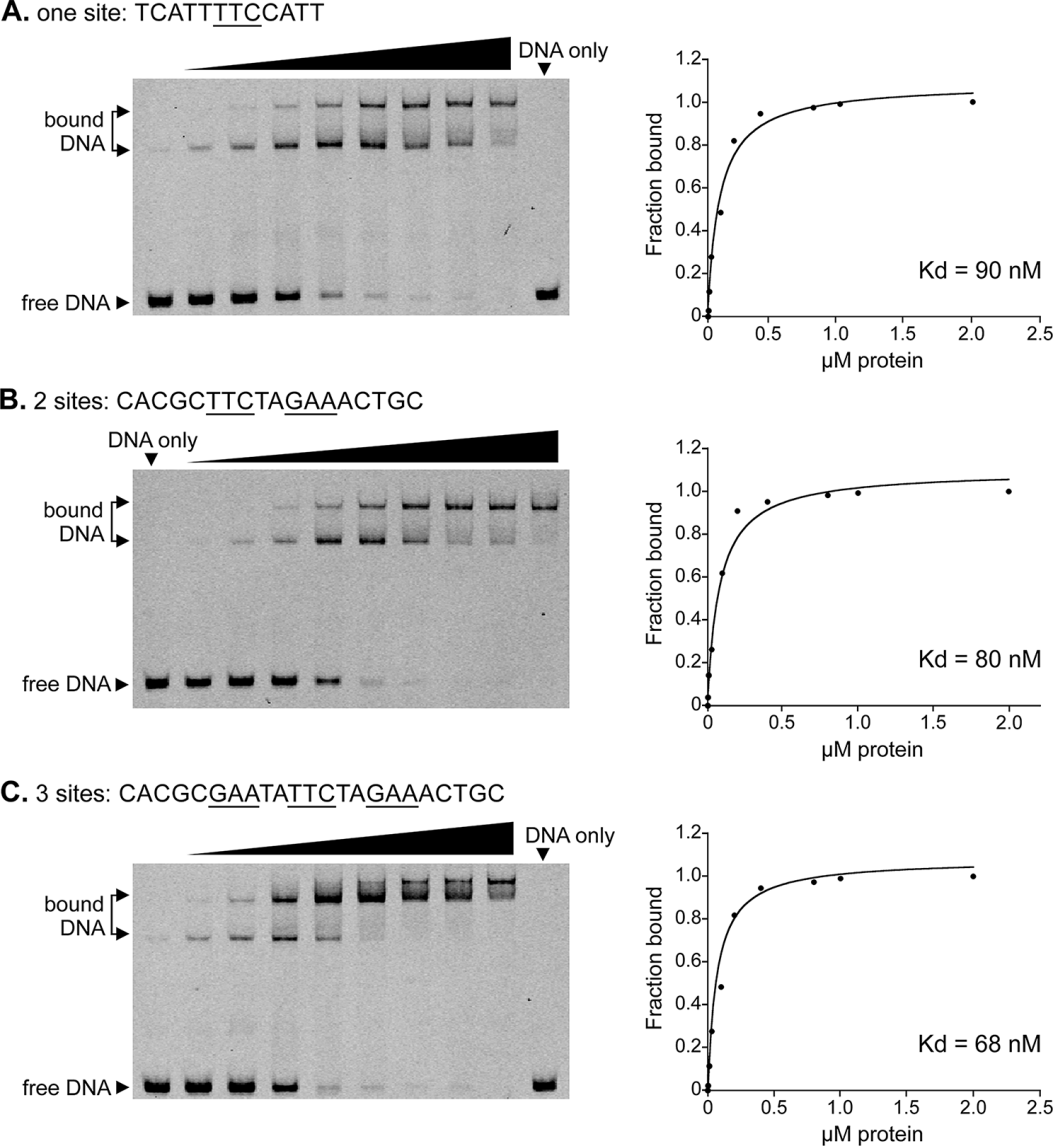
Binding of HSF protein PITG_04701 to targets with one, two, or three TCC/GAA units. These EMSA assays used 20 nM of Cy5-labeled double-stranded oligonucleotides bearing the indicated sequences flanked by 14–17 nt of random DNA, and 0 to 2000 nM of PITG_04701 (triangle). The fraction of bound DNA was determined by densitometry of the gels and used to calculate K_*d*_. The portion of the protein tested here as well as on the PBMs (Table S1) includes the coiled-coil domain which is believed to enable oligomerization. Full-length gels are presented in Figure S5

Although HSF proteins in other kingdoms often form oligomers that can bind three or more of units of TTC or its reverse complement [59, 68, 69], our motifs usually contained only one or two units. This likely reflects the tendency of the PBM approach to underestimate the width of binding sites [65]. We addressed this by using EMSA to measure the binding of PITG_04701 to one, two, or three copies of TTC (Fig. 3). Dissociation constants calculated from those assays (Fig. 3, right) indicated that binding to the trimeric DNA site was stronger than to the dimer or monomer. Binding to the trimer was not as strong as that reported for human HSF1 [68], but this might be explained by variation in the methods employed (EMSA versus fluorescence polarization) or our use of a higher pH (7.9 versus 7.5) which may suppress oligomerization [70]. Nevertheless, the ability of PITG_04701 to bind diverse sites may enable graded control of transcription. Consistent with this, we detected both dimeric, trimeric, gapped, and ungapped target motifs in the promoters of *P. infestans* genes encoding Hsp70, which in other species are often regulated by HSF factors [71].

PITG_04701 contains a coiled-coil domain (i.e., hydrophobic heptad repeats) near its DNA binding-domain. This configuration occurs in animal, yeast, and plant HSFs where the repeats enable oligomerization [72–74]. The occurrence of a coiled-coil domain in PITG_04701 is consistent with the multiple large, retarded bands observed in EMSA (Fig. 3), although some of the bands might be due to individual proteins binding separate motifs. The presence of this putative oligomerization domain is also consistent with the protein’s optimal trimeric binding site. Due to the universal presence and functional importance of coiled-coil domains in plant, fungal, and animal HSF proteins, we were surprised to discover that only 7 of the 17 *P. infestans* proteins (PITG_03306, 04700, 04701, 05353, 06935, 08199, 15654) had coiled-coil domains based on the six prediction tools within Waggawagga and a newer neural network method, DeepCoil [41, 42]. The absence of coiled-coils did not appear to be due to errors in gene models based on the alignment of the genes to RNA reads and since orthologs mined from other oomycete genomes had similar structures.

RNA-seq data revealed diverse patterns of expression of the HSF genes, with those binding similar motifs often displaying distinct transcriptional profiles and vice versa (Fig. 2B). All 10 genes were upregulated in at least one life-stage or growth condition, especially in one of the spore stages. For example, PITG_04694 and PITG_04701 were expressed primarily in zoospores. Even though their genes reside within 50 kb which suggests evolution from a common ancestor, their protein sequences did not cluster in the phylogram and their DNA binding motifs are distinct. Similarly, while PITG_03306 and PITG_22459 were transcribed primarily during late tuber infection, they did not cluster in the tree and had distinct binding sites. In contrast, PITG_11760 and PITG_22459 clustered and bound similar motifs but had divergent expression patterns although both were upregulated during mating.

In other species, subsets of HSF genes are transcribed constitutively, developmentally-regulated, or induced by stresses including heat, starvation, and reactive oxygen [75]. It was therefore notable that PITG_11760 and PITG_20378 were induced in our minimal media, a near-starvation condition which supports very poor growth. We also discovered that PITG_05353, PITG_08236, and PITG_11760 are upregulated by hydrogen peroxide by mining data from a prior study [76].

### bZIP family

**Fig. 4:**
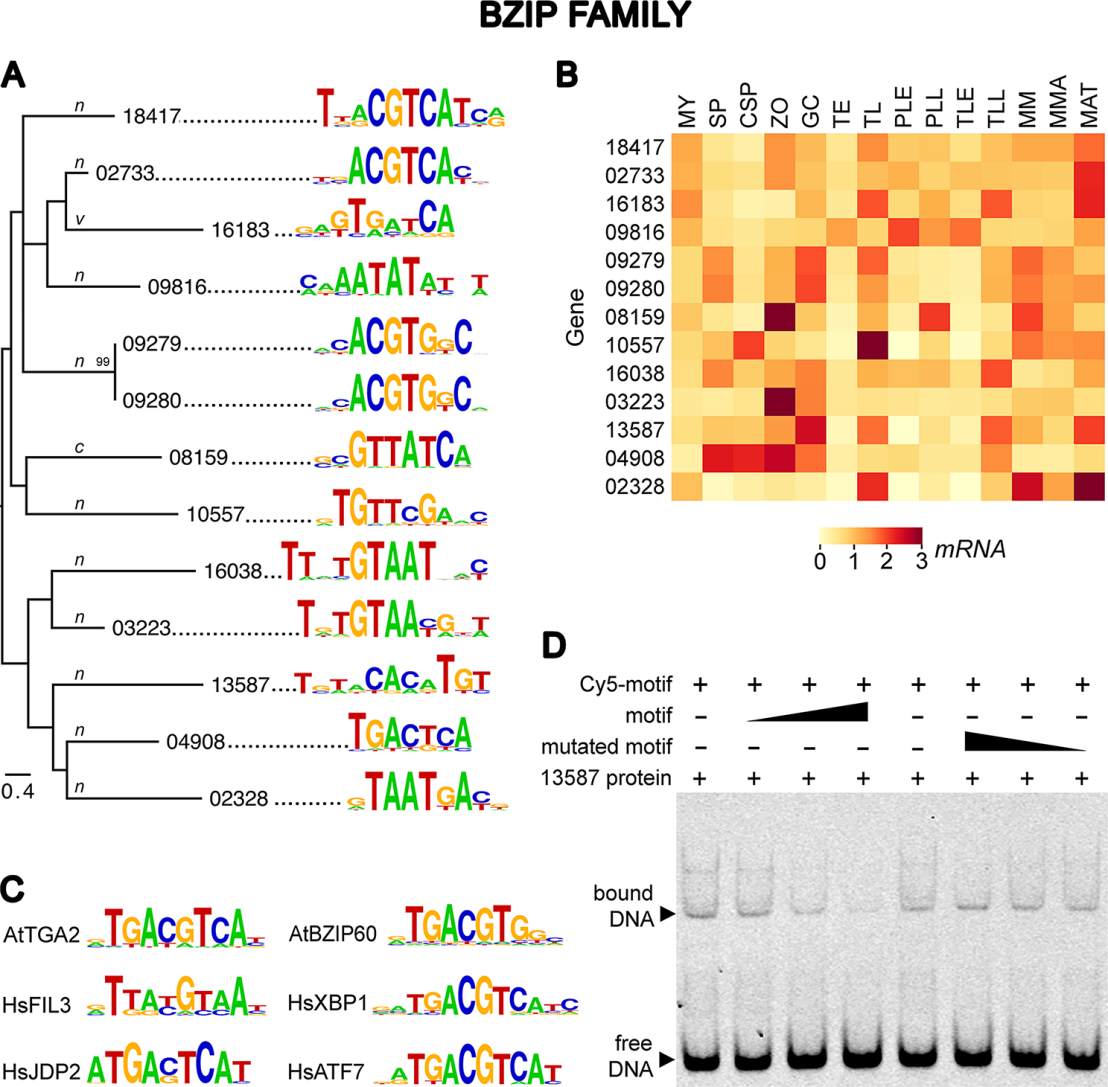
DNA binding specificities of bZIP family. The organization of panels **A**-**D** are the same as in Fig. 2 except that the unlabeled competitors used in EMSA were at 1, 10, 50-fold the concentration of Cy5-labeled probe. The single-letter code on the branches of the phylogenetic tree indicates whether the TFs were the Asn (n), Cys (c), or Val (v) types. The codes for the RNA samples in panel B are shown in Fig. 2. A full-length gel is presented in Figure S5

This group includes members with a canonical DNA-binding domain and those in which an evolutionarily conserved Asn corresponding to residue 235 of *Saccharomyces cerevisiae* GCN4 is substituted by Cys, Val, or Tyr [11, 77]. Thirty bZIPs with detectable transcription were identified and used for PBM analysis including 17, 7, 5, and 1 in the Asn, Cys, Val, and Tyr categories, respectively. DNA motifs were predicted for 11 of the 17 Asn bZIPs (Fig. 4A). In contrast, motifs were identified for only one of the seven Cys types and one of the five Val forms. The predicted motif for PITG_13587 was validated by EMSA (Fig. 4D). bZIP proteins bind DNA as dimers; we speculate that some proteins that did not yield motifs may be obligate heterodimers.

The position of a bZIP on the phylogenetic tree often correlated with its DNA-binding specificity. Four of the six bZIPs in the largest clade, ranging from PITG_18417 to PITG_09280, bound sequences with a palindromic ACGT core. This motif also occurs within many targets of bZIPs from *A. thaliana* and humans (Fig. 4C). However, binding preferences lacking an ACGT core were predicted for about half of the *P. infestans* bZIPs, representing the lower clades of the tree.

About 85% of the bZIPs appeared to bind palindromes, which is expected since this family typically acts as dimers [7]. For example, the palindrome recognized by PITG_09816 was ATATAT. Others bound gapped palindromes, such as PITG_04908 (TGACTCA). Although palindromes were not evident in the consensus logos of several other bZIPs, individual 8-mers from their PBM data were often palindromic (Fig. S3). Examples are PITG_16038 and PITG_02323 (GTAATTAC), PITG_10557 (GTTCGAAC), and PITG_16183 (CATCGATG).

Transcripts of about three-quarters of the bZIPs were induced in at least one stage of the life or disease cycles (Fig. 4B). However, fewer bZIPs than HSFs had dynamic changes in mRNA levels during mycelial-spore transitions while more bZIPs were upregulated in hyphae from minimal media compared to rye-sucrose media. Most bZIP exhibited low relative mRNA levels in the early (biotrophic) stages of tuber and leaf infection. Interestingly, most other TF families including HSFs showed this same pattern.

Continuing another trend seen with the HSFs, the DNA-binding preference of a bZIP and its transcription profile were not always correlated. For example, PITG_18417 and PITG_02733 bound similar motifs and had similar expression profiles. In contrast, PITG_16038 and PITG_03223 had different patterns of expression despite having similar binding preferences.

### Myb family

**Fig. 5:**
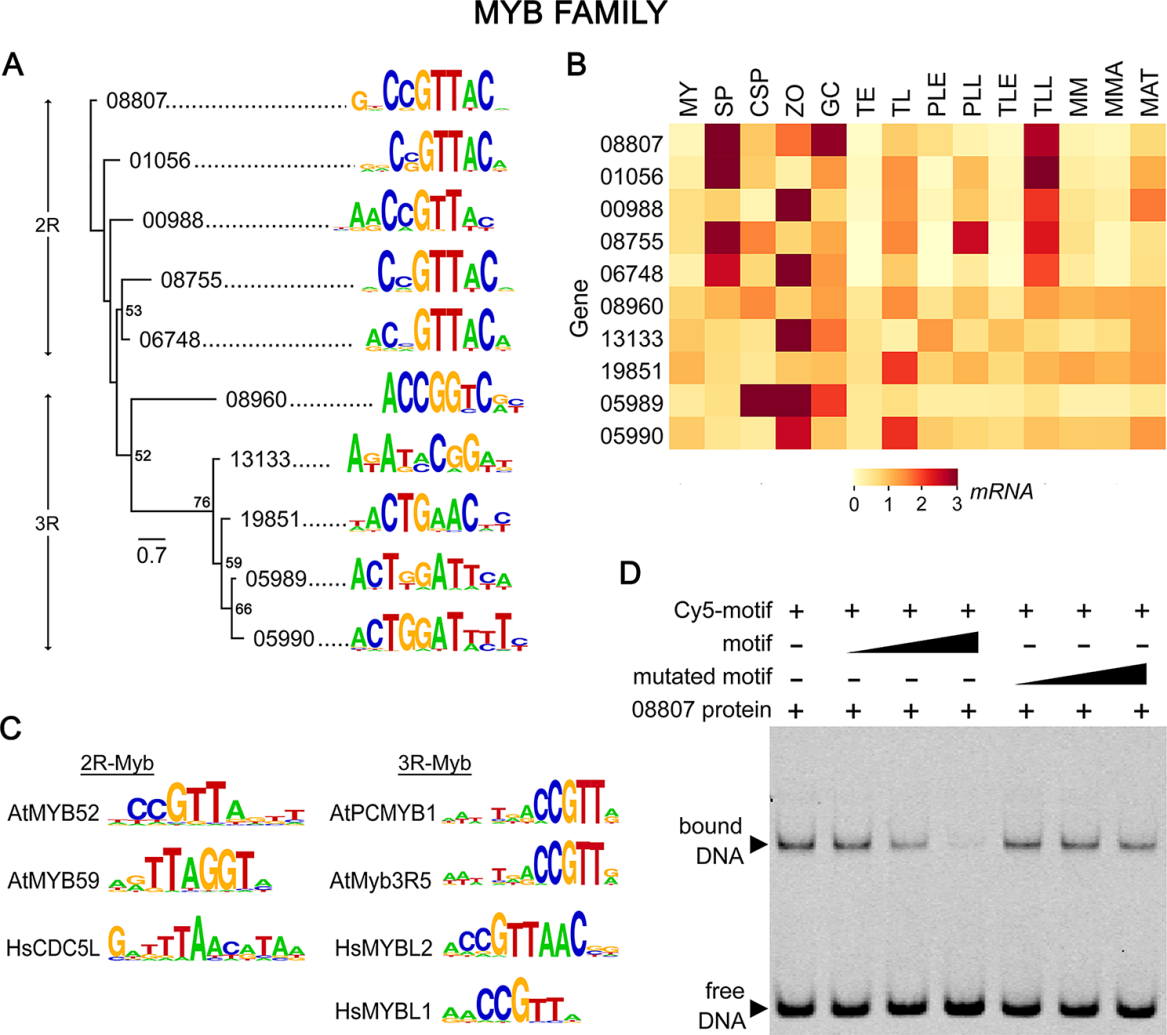
DNA binding specificities of MYB family. The organization of panels **A**-**D** are the same as in Fig. 2. The clades in the phylogenetic tree are marked to denote the 2R-Myb and 3R-Myb types. A full-length gel is presented in Figure S5

As in other eukaryotes, *P. infestans* encodes proteins with one, two, or three Myb DNA-binding domains which are named 1R, 2R, and 3R-Myb proteins, respectively. Since many proteins with single Myb domains lack sequence-specific TF activity [56, 78], we focused our PBM studies on the 2R and 3R proteins. DNA binding specificities for six of seven 2R and five of nine 3R types were obtained (Fig. 5A).

The DNA motifs for the 2R and 3R groups were distinct (Fig. 5A). The 2R members all bound sequences sharing CCGTTAC, which resembles the targets of human and *A. thaliana* 2R-Mybs (Fig. 5C). This target was confirmed for PITG_08807 by EMSA although a tendency for nonspecific binding was evident since the unlabeled oligonucleotide was only about five times more effective as a competitor than one with a mutated motif (Fig. 5D). Non-specific binding of Myb proteins from other kingdoms has also been described [79]. In contrast to the 2R-Mybs, the five 3R types bound more divergent sequences although three shared an ACTG motif. None of the targets resembled those bound by human or *A. thaliana* 3R-Mybs (Fig. 5C).

Our RNA-seq data revealed distinct patterns of expression between and within the 2R and 3R-Myb subfamilies (Fig. 5B). One difference was that four of the five 2R-Mybs, but no 3R form, were upregulated strongly in sporangia. Continuing a trend seen with other TF families, members within both the 2R and 3R groups that had similar DNA-binding domains and target motifs often had different transcriptional profiles. For example, PITG_05989 and PITG_05990 bound similar DNA motifs, but mRNA levels of the former were upregulated in sporangia chilled to initiate zoosporogenesis (CSP) while the latter was not upregulated until after zoospores were released (ZO). Also, only PITG_05990 was induced during late tuber infection. Another continuing trend was that transcript levels were typically low during early plant infection, especially for the 2R-Mybs.

### C2H2 zinc finger family

Twenty-two proteins were identified with high-confidence as C2H2 TFs from *P. infestans* based on the presence of two or more C2H2 DNA-binding domains. An additional 50 proteins were defined as low-confidence hits since they either contained sequences seemingly inconsistent with TF activity such as retroelement domains or bore only a single C2H2 domain, which is normally insufficient for binding DNA [80, 81]. It is possible that some of the latter might have affinity for DNA in the presence of other structural elements as seen with plant Superman proteins [81]. No protein resembled the related DOF single-domain TFs of plants [82].

**Fig. 6:**
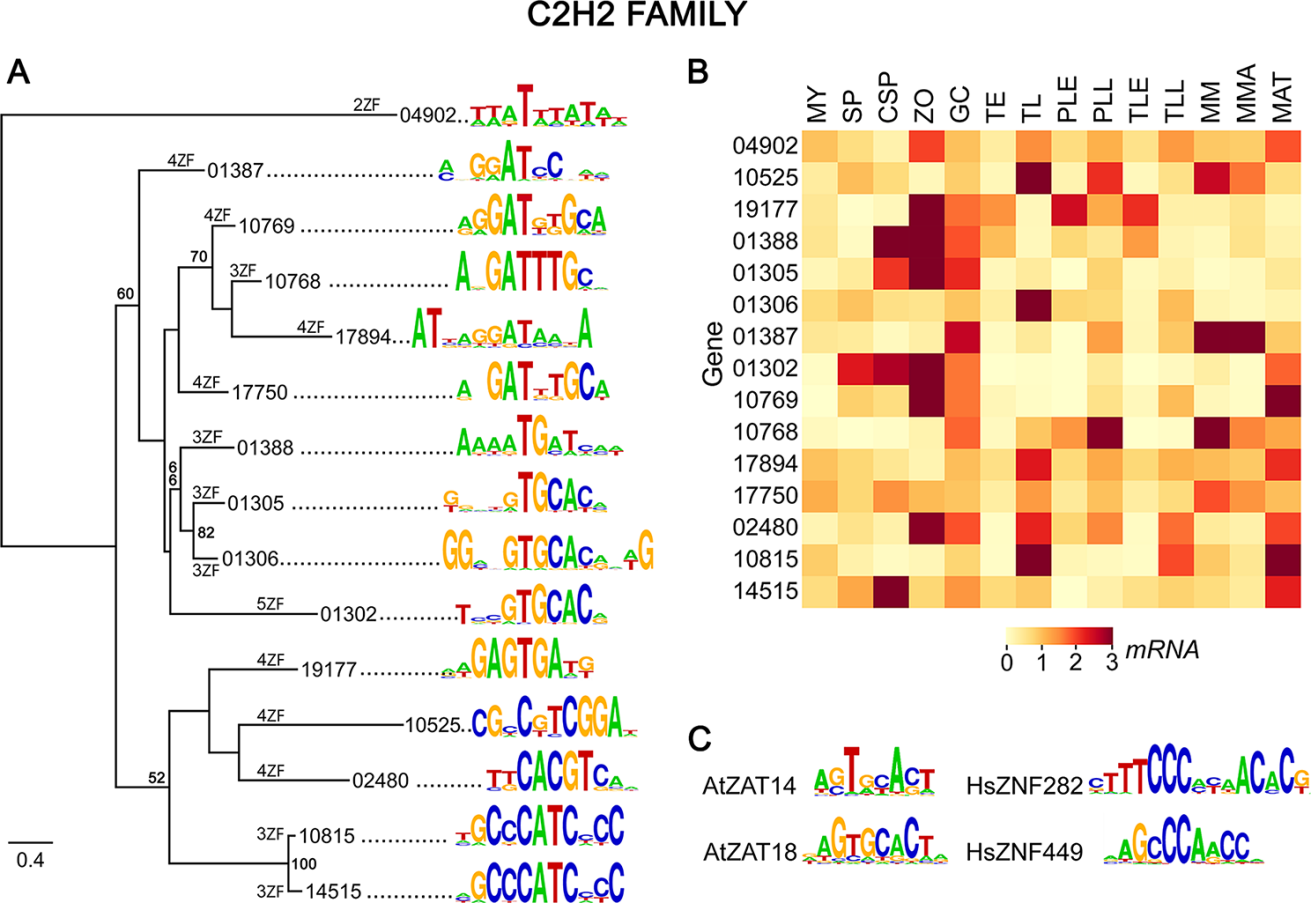
DNA binding specificities of C2H2 zinc-finger family. The organization of panels **A**-**C** are the same as in Fig. 2. The number of zinc fingers in each protein is marked on the branches in panel A

Our PBM studies were limited to the high-confidence hits, and resulted in the definition of binding preferences for 15 proteins (Fig. 6A). These exhibited more variation in binding preference than seen within the other TF families. Nevertheless, there was some congruence between the motifs bound by some C2H2 proteins and their position in the phylogenetic tree. For example, the clade containing PITG_01388 and PITG_01306 all bound sequences containing GTGCAC, while the motifs for clustered proteins PITG_10815 and PITG_14515 both had a GCCCATC core. The latter resembles the sites bound by human ZNF282 and ZNF449 but considering the diversity of the human family this may not imply an evolutionary relationship since there was little similarity between the amino acid sequences of the oomycete and human DNA-binding domains. Similarly, the motif predicted for PITG_01305 resembled the targets of *A. thaliana* ZAT6 and ZAT18.

Binding sites within the plant and animal families are also diverse due to variation in the sequence, number, or spacing of their zinc fingers [65, 83]. Each *P. infestans* C2H2 protein contained an average of 3.5 zinc fingers, ranging between two and five. This is similar to the number in other oomycetes, but less than the nine found in the average human C2H2 protein [83] and more than the average of two fingers in *A. thaliana*, fungi, and members of the SAR (stramenopile-alveolate-rhizaria) supergroup.

As with other TF families, many C2H2 genes had mRNA levels that increased in zoospores and germinated cysts (Fig. 6B). However, compared to other families, fewer genes were upregulated in sporangia and more rose during mating, such as PITG_10815 and PITG_14515. Interestingly, although these two clustered in the tree and bound similar motifs, only PITG_14515 was expressed strongly in chilled sporangia and only PITG_10815 in late tubers, providing another example of possible subfunctionalization. Slightly more C2H2 genes were expressed during early infection than seen for other TF families.

### Homeodomain family

**Fig. 7:**
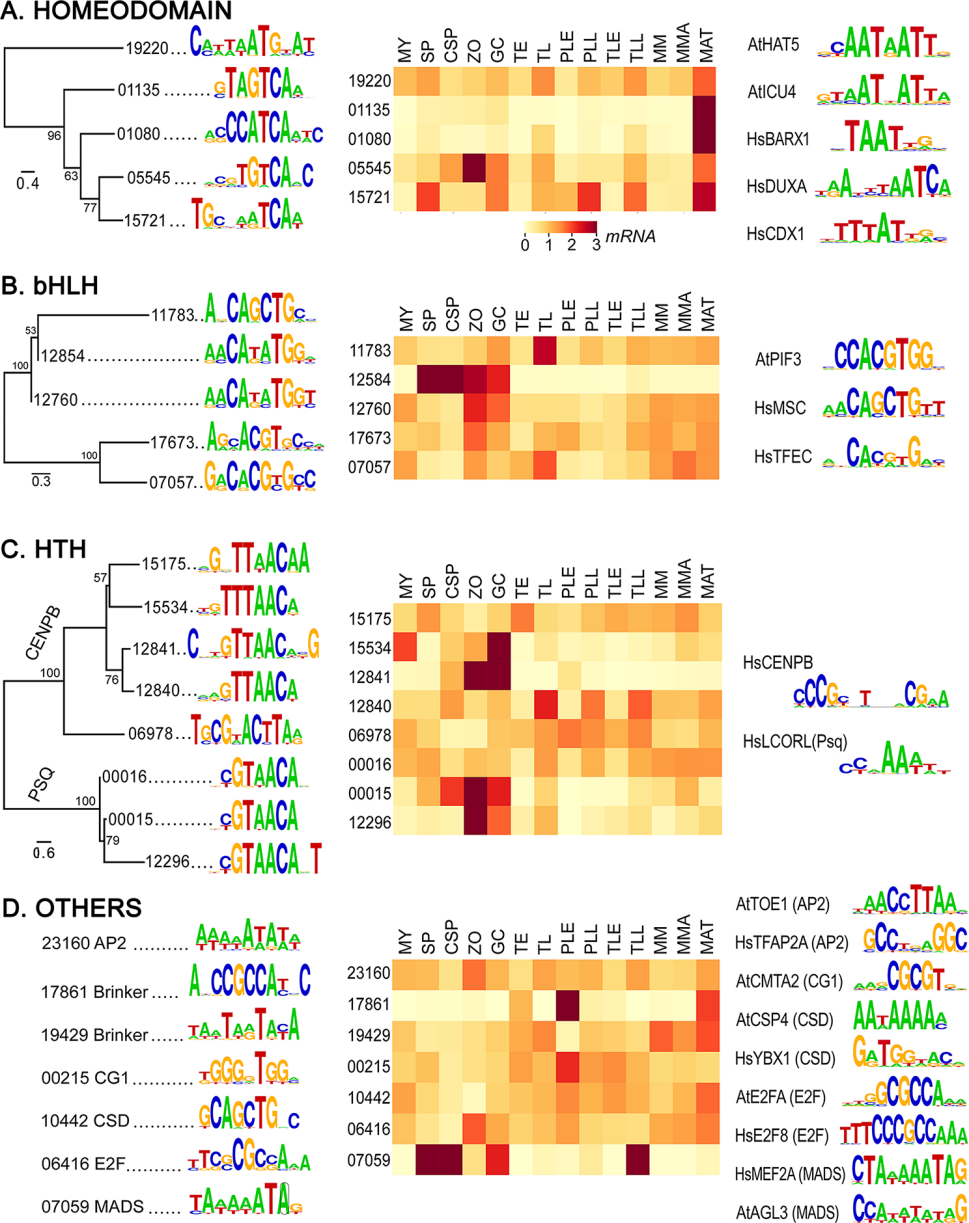
DNA binding specificities of homeodomain, bHLH, HTH, and other TF groups, showing phylogenetic trees and predicted motifs (left), RNA level heatmaps (center), and motifs bound by *A. thaliana* and human orthologs (right, with At and Hs prefixes)

Binding preferences were determined for all five members of this family (Fig. 7A, left). Their motifs were largely dissimilar although four of the five predicted targets contained TCA. While most human and *A. thaliana* homeodomain TFs bind AT-rich sequences, this was observed only for PITG_19220. That protein contains three homeodomains which were tested separately, but only the N-terminal domain yielded a positive result.

A striking feature of the expression profiles of the *P. infestans* genes was that all were expressed highly during mating (Fig. 7A, center). This was especially true for PITG_01080 and PITG_01135. Despite this similar pattern, their predicted DNA targets only shared a TCA motif. None of the top twenty 8-mers bound by each protein on the arrays were in common.

### Basic Helix-Loop-Helix (bHLH) family

DNA targets were identified for all five proteins in this group, with similarities in their motifs congruent with locations of the proteins in the tree. Consistent with data from other species that bHLHs bind as dimers, the DNA targets all contained the gapped palindrome CANNTG (Fig. 7B, left). This is also within the targets of the representative bHLHs from *A. thaliana* and human, which have been described as E- (CANNTG) and G- (CACGTG) boxes, respectively (Fig. 7B, right) [65, 84].

The *P. infestans* genes displayed diverse patterns of expression despite having similar DNA-binding preferences (Fig. 7B, center). For example, PITG_11783 was transcribed at similar levels in most tissues except for tubers where its mRNA levels were high. In contrast, PITG_12584 was expressed primarily in the asexual spores.

### Helix-Turn-Helix (HTH) families

The HTH domain occurs in two families of DNA-binding proteins, CENPB and Pipsqueak (Psq). While Psq proteins regulate protein-coding genes, human CENPB participates in assembling centromeres and repressing transcription of non-coding sequences [85, 86]. A relative of CENPB (but not Psq) may exist in *A. thaliana*, but its binding sites are unknown [87].

PBM analysis identified binding motifs for five of seven CENPB and three of four Psq proteins from *P. infestans* (Fig. 7C, left). Despite functional differences between CENPB and Psq, nearly all of their predicted binding sites shared a TAACA motif. This does not occur in the targets of human CENPB or Psq (Fig. 7C right). Despite the similar binding preferences of the *P. infestans* proteins, each group displayed distinct and diverse transcriptional profiles (Fig. 7C, center). For example, PITG_12296 and PITG_00015 exhibited spore-associated expression patterns while PITG_00016 was more constitutive.

While human CENPB proteins bind a 17 bp centromeric motif called the CENPB box (Fig. 7C, right), we determined that the GTTTAAC and GTTAAC motifs bound by the proteins from *P. infestans* are not enriched at its centromeres. Unlike the human CENPB box, the *P. infestans* motifs are palindromes which suggests that its proteins have a distinct function.

### Other families

DNA targets were also determined for the small Brinker, E2F, CSD, AP2, CG1, CSD, E2F, and MADS-box families which have five, three, two, two, one, one, and one members, respectively (Fig. 7D, left). In several cases the targets resembled those of *A. thaliana* and human proteins (Fig. 7D, right). For example, MADS-box proteins from all three species bound AT-rich sequences, while E2F proteins all targeted motifs bearing CGCCA. In contrast, the binding sites of the *P. infestans* AP2, CG1, and CSD proteins were dissimilar to those from the human and plant. We are unaware of a known binding site for a human Brinker, but the binding specificity of PITG_17861 resembles the GC-rich targets of Brk from *Drosophila melanogaster* [88].

A range of expression patterns were observed for the *P. infestans* TFs in this section. Most showed moderate mRNA levels in most tissue samples, while PITG_17861 (Brinker) and PITG_07059 (MADS) were strongly stage-specific. For example, PITG_07059 was upregulated strongly in sporangia.

### Chromatin immunoprecipitation confirms the predicted MADS-box target

**Fig. 8:**
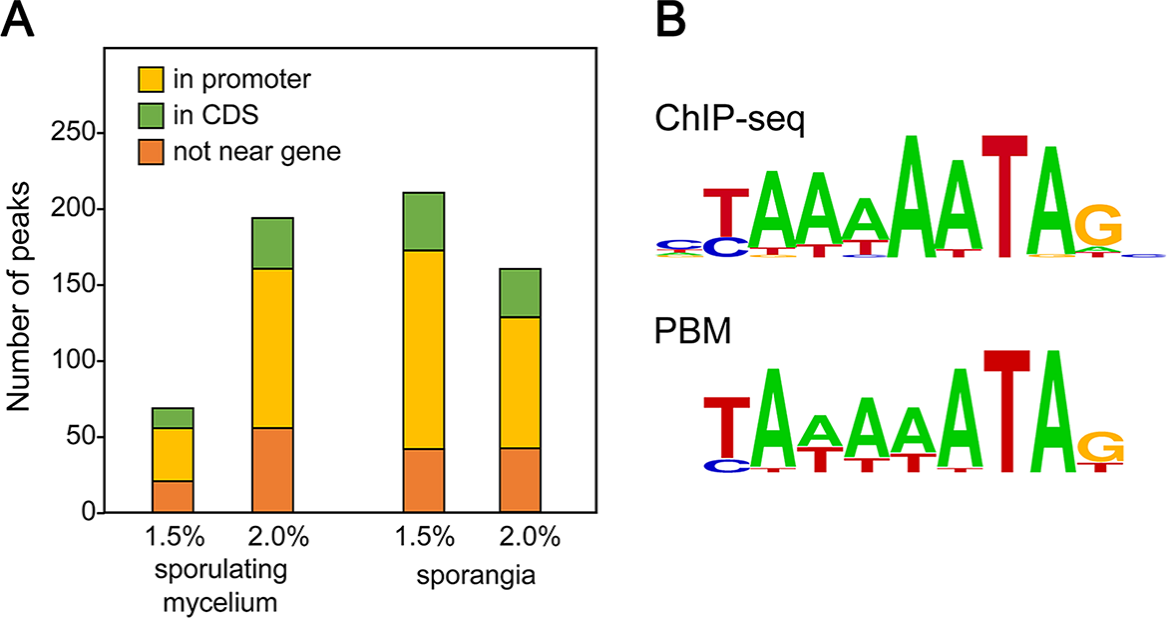
Targets of MADS-box protein determined by ChIP-seq. (**A**) Number and genomic context of peaks in chromatin from sporulating mycelia and sporangia crosslinked with 1.5% and 2% formaldehyde. Locations include coding sequences (CDS), promoters, and sites distant from genes. (**B**) Logo plots representing motifs enriched in promoter region based on the ChIP-seq and PBM data

Although a prior study in a mammalian system concluded that TF binding preferences identified by ChIP-seq matched in vitro binding results [37], we chose to also test this with an oomycete TF. We examined PITG_07059 since a satisfactory antibody had been generated as part of a prior study [12]. Due to the gene’s sporulation-associated expression pattern, samples for ChIP-seq were prepared from both sporulating mycelia and purified sporangia. This led to the identification of 259 and 367 peaks across chromosomes for the two tissues, respectively (Fig. 8A). These reside predominantly in promoters. The sequence over-represented in those peaks matched that obtained from the PBM (Fig. 8B).

### Enrichment analysis links DNA targets to expression profiles

To better understand the roles of the TFs, we calculated whether their motifs showed biased representation in promoters with specific patterns of activity. To accomplish this, we developed lists of genes having mRNA levels that were 10-fold higher in each tissue compared to nonsporulating mycelia in rye-sucrose broth; genes higher in nonsporulating mycelia were identified by comparison to sporangia. Then, matches to the motifs were identified using FIMO with the default *P*-value threshold of 10^− 4^ [52]. We hypothesized that targets of TFs that upregulate genes in a specific tissue would be over-represented in the relevant promoter set, while under-represented sites might bind repressors or stimulate transcription in other stages. A second hypothesis is that sites binding general activators would show little bias across the promoter sets.

**Fig. 9:**
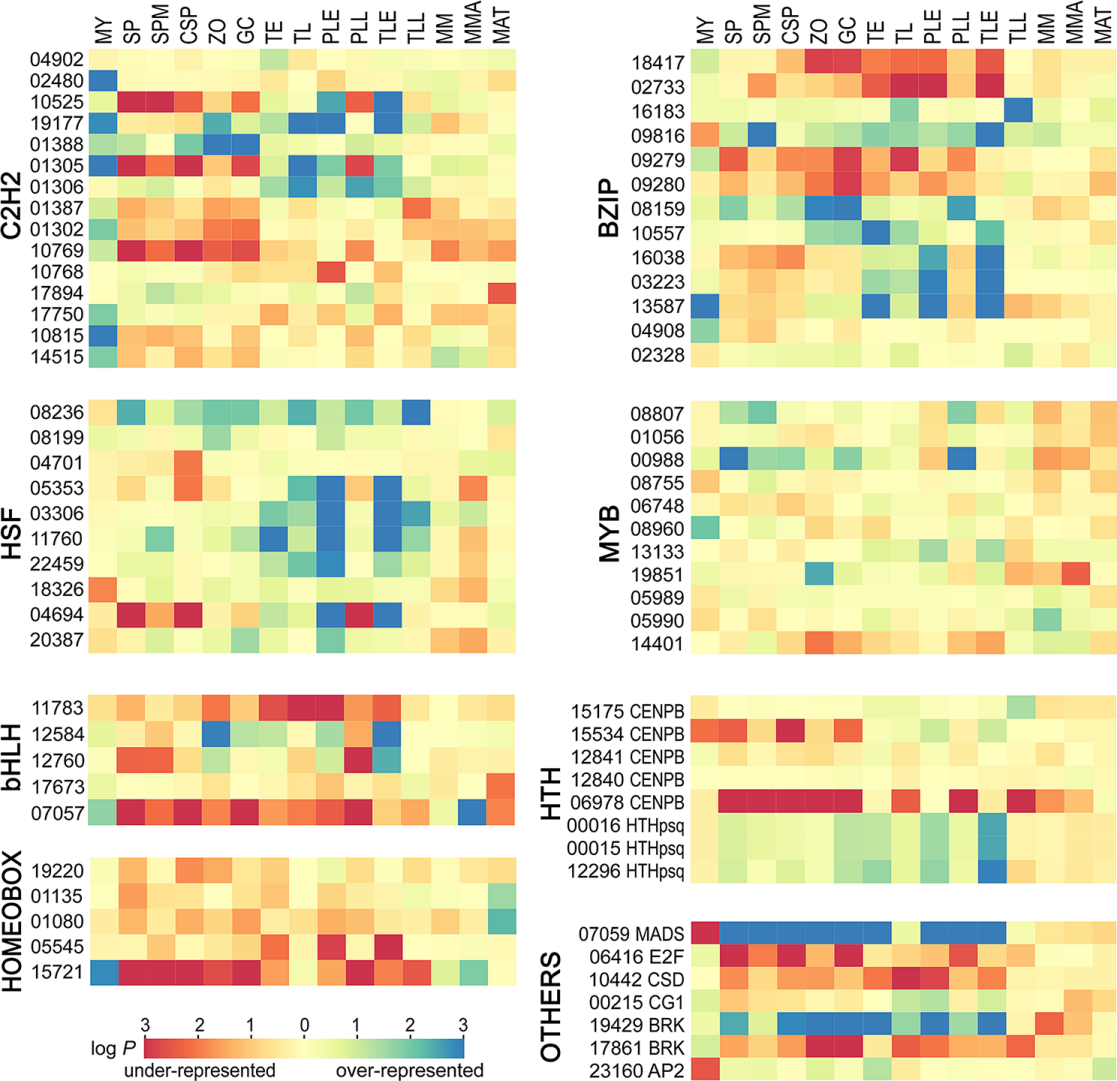
Enrichment analysis of TF binding sites in promoters displaying different patterns of activity. The heatmaps display log_10 _*P*-values associated with over- or under-representation (blue and red, respectively) in promoters upregulated > 10-fold in the indicated tissues when compared to nonsporulating mycelia, except that nonsporulating mycelia (MY) was compared to sporangia (SP) and the mating culture (MAT) was compared to the mean of the parents grown separately. The tissues are the same as in Fig. 2 with the addition of SPM, which represents the entire tissue (sporangia and mycelia) from 5-day cultures

As shown in Fig. 9, 68 of the 73 TF motifs were over-represented (blue) or under-represented (red) in one or more promoter sets, based on a *P*-value threshold of 0.05. The most common patterns included over-representation in genes upregulated during plant infection, mycelial growth, or spore development. However, the trends varied between and within the TF families. For example, while about half of the C2H2 binding sites were enriched in genes expressed more in nonsporulating mycelia, this was true for only one bZIP and one homeodomain target.

Supporting our hypothesis that over-represented motifs would be linked to stage-specific transcription, the binding site for the MADS-box TF was overly abundant in promoters of genes upregulated in sporulating hyphae and sporangia (Fig. 9, lower right panel). This protein was proven earlier to regulate many sporulation-induced genes [12]. Its binding site was also abundant in promoters upregulated in many of the plant infection samples. In the late infection timepoints, this might be attributed to the occurrence of sporulation. However, over-representation of the motif in the early timepoints might instead reflect the involvement of a different TF with a similar binding site, since sporulation initiates only near the end of the disease cycle.

We extended our search for motifs in co-regulated gene sets to search for potential *cis*-regulatory modules (CRMs). These are defined as clusters of binding sites for distinct TFs that combine to dictate a pattern of expression, and are common in the promoters of plants and animals [89]. CRMs are typically identified by searching for over-represented combinations of TF binding sites within a genome [90]. We therefore searched total *P. infestans* promoters and subsets upregulated at each stage of the life cycle using MCAST [91] and custom scripts using outputs from FIMO [52]. After eliminating gene families in which promoter and coding sequences of members were nearly identical, no over-represented motif combinations (potential CRMs) were identified.

### Functional analysis of a motif confirms its predicted function

**Fig. 10:**
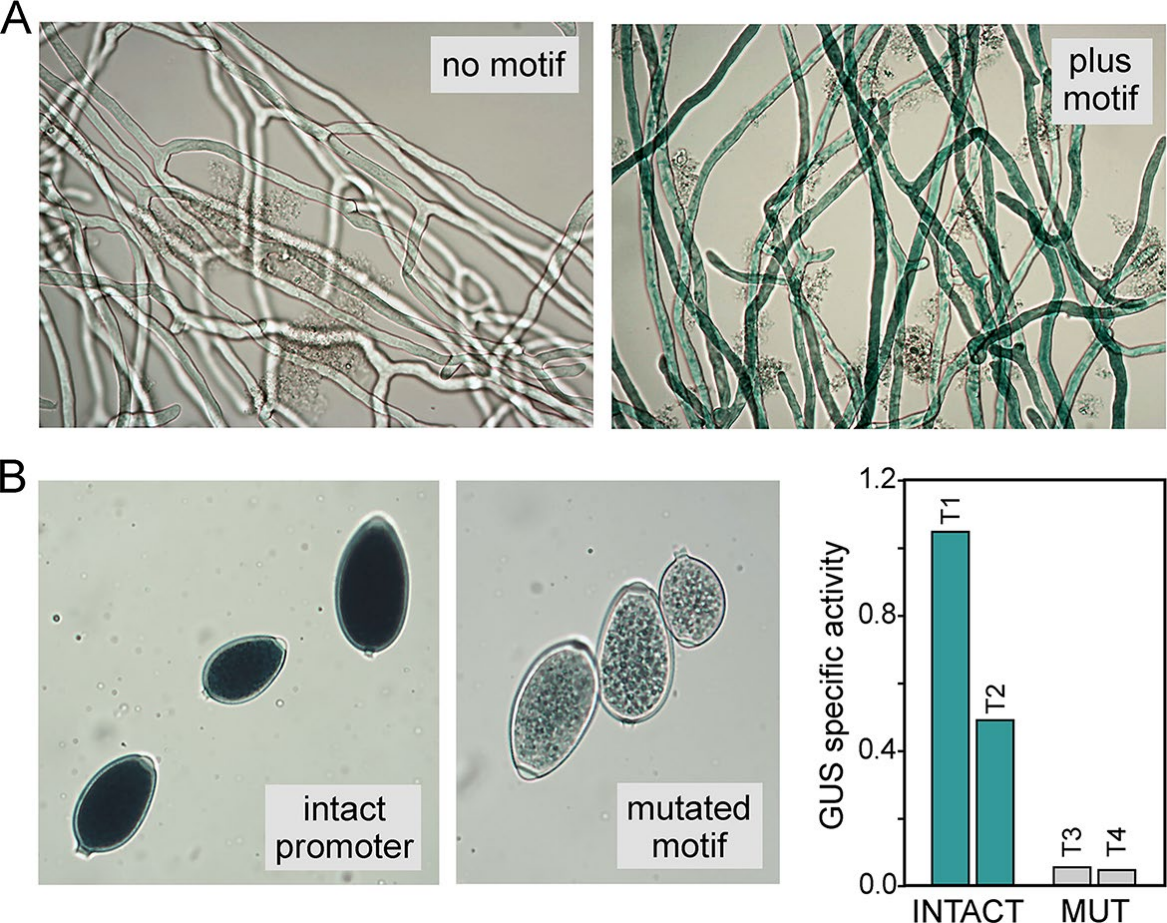
Evaluation of the PITG_04701 binding site in *P. infestans* transformants. (**A**) Tests using the minimal *NifS* promoter fused to GUS. The left panel shows a representative transformant with the unmodified minimal promoter, subjected to histochemical staining. As described previously [28], this partial promoter does not drive expression in any life stage. However, expression was detected when the PITG_04701 motif was inserted upstream of the minimal promoter. Similar results were obtained with four independent transformants. (**B**) Assays using the GUS gene fused to the promoter of PITG_20221, which contains the PITG_04701 motif. Indicated are a representative transformant containing the intact promoter (left) and one in which the PITG_04701 binding site was mutated (right). Similar results were obtained with four independent transformants. While deleting the PITG_04701 motif did not prevent induction of the gene, the mutation strongly repressed expression. The graph shows quantitative data from a fluorometric assay using sporulating hyphae from two transformants with the intact promoter (T1, T2) and two with the mutation (T3, T4. Due to position and transgene copy number effects, transformants obtained with the same plasmid (e.g., T1 and T2) are not expected to yield the same level of expression

In the analysis shown in Fig. 9 several motifs lacked a strong association with any stage-specific pattern of expression. We hypothesized that such motifs bind general activators. Prior studies have shown that adding the DNA target of a general activator to a minimal promoter often stimulates transcription, while its elimination from a full promoter reduces expression [92]. This was tested using the motif for HSF PITG_04701 which was not over-represented significantly in any promoter set. As shown in prior studies [28] and repeated here, the *NifS* minimal promoter does not drive the β-glucuronidase (GUS) reporter when transformed into *P. infestans* (Fig. 10A). However, expression resulted when the motif bound by PITG_04701 was added 5’ to the *NifS* sequences. Also as hypothesized, transcription was impaired by mutating the motif in a full promoter. While the intact promoter drove robust expression of the reporter, a weak signal resulted when the motif was mutated. This was shown initially using histochemical staining and then confirmed by a quantitative assay (Fig. 10B). As might be expected for a general activator, PITG_04701 is well-expressed in all stages, ranking for example in the 77% percentile of genes in mycelia. The gene is upregulated in zoospores, which might relate to the increase in expression of general housekeeping genes that occurs when their cysts germinate [22].

## Discussion

We have defined the DNA binding preferences of 73 TFs from *P. infestans* representing 14 of its 22 families and associated many of those TFs with patterns of gene expression during the life cycle. Several binding sites were verified by EMSA or ChIP-seq. The latter represents its first application to an oomycete TF. Combined with RNA-seq resources [22–24, 39], methods for blocking gene activity through homology-based gene silencing or editing [51, 93], a chromosome-scale genome assembly [19], and emerging data on nucleosome occupancy [94] the prospect of revealing the networks that regulate growth, development, and pathogenesis are now much improved.

Our success rate of 59% for proteins tested on the PBMs compares favorably with results from other organisms [37, 58]. The failures might be due to the absence of a cofactor or post-translational modification, a requirement for heterodimerization [95], or poor folding of the proteins. Some failures might be related to the fact that we did not test the whole protein, although prior studies indicated that full-length TFs and their isolated DNA-binding domains nearly always bound similar sequences [62]. In both the current and prior studies, a strong correlation was observed between binding data from in vitro studies using DNA-binding domains alone and ChIP-seq data targeting the native protein in vivo [37].

Reflecting on some causes for failures on the PBMs may provide insight into the biology of *P. infestans.* For example, yielding poor results were the bZIPs with novel amino acids (Cys, Val, or Tyr) in their DNA-binding domains. It is possible that these only associate with DNA as heterodimers. Alternatively, rather than binding DNA directly their cellular function might entail blocking the attachment of the canonical Asn bZIPs to DNA by forming heterodimers. Another possibility is that DNA binding by the Cys types may depend on that residue’s oxidation state, which would be consistent with our discovery that those bZIPs help defend against oxidative stress [11, 96].

Our phylogenetic analyses revealed that TFs within a cluster often had similar binding specificities, in line with findings from other taxa [37, 97]. Cases where clustered TFs bound distinct sequences could often be explained by small variations in their DNA-binding regions. PITG_05989 and PITG_19851, for example, differ in one of the eleven residues in each of their R1 and R2 Myb domains that are thought to bind DNA [98].

TFs in a family that bound the same motif often had distinct patterns of expression consistent with subfunctionalization after gene duplication. This transcriptional divergence may have resulted from incomplete duplication of the promoter, small mutations, or acquisition of a new regulatory site [99, 100]. For functionally equivalent paralogs, a new expression profile might serve to fine-tune mRNA levels through the life cycle, giving a basis for retaining the duplicated gene. The new pattern might also be beneficial if changes outside the DNA-binding domain conferred a new function. For example, while C2H2 proteins PITG_10815 and PITG_14515 target the same DNA sequence and have similar DNA binding domains, their 200-amino acid C-termini lack similarity. These might associate with different proteins or cofactors as described for paralogs in other systems [101, 102].

Non-paralogous TFs also often had similar predicted binding specificities or targets with staggered overlaps. One such example involves the MADS-box protein PITG_07059 and Brinker protein PITG_19429. Such proteins may compete for binding, switching the transcription pattern of a target gene [103]. The interaction may also be synergistic, enhancing transcription through an assisted loading model [104].

Prior studies showed that TF orthologs within a taxonomic group such as plants, animals, or fungi often have similar binding specificities [37, 88, 105]. However, few other than HSFs have been shown to bind similar targets across kingdoms [106]. Our data have thus extended knowledge of binding site conservation across long evolutionary distances by examining oomycetes, which lack taxonomic affinity with traditional model organisms [10]. Members of five *P. infestans* families (bZIP, E2F, HSF, Myb, MADS-box) targeted motifs resembling those of relatives from plants and/or humans. Sometimes the sequences were nearly identical, as with the human and *P. infestans* MADS-box proteins. More often there was only partial overlap as with a subset of the *P. infestans* bZIPs where only an ACGT core was shared across kingdoms. Interestingly, while the *P. infestans* HSF proteins bound the canonical TTC motif, a majority lacked an obvious coiled-coil domain that is central to their function in other kingdoms [72–74, 107].

Besides assigning binding sites to the *P. infestans* TFs, many were linked to specific patterns of transcription based on their relative representation in promoters. While similar approaches have been employed in other species [65, 108], the method can be challenging. Statistical significance may be hard to achieve due to noise from binding site degeneracy or if a TF regulates only a few genes. Another complication is that an expression pattern may be determined by several TFs operating independently, multiple regulators working in concert through heterodimerization or as part of a TF cascade, or TFs acting *in trans* by co-opting cofactors. Thus, additional strategies will be needed to link many TFs to their cellular targets.

## Conclusions

The databases of transcription factors and their binding specificities yielded by this study are foundations for future explorations of the evolution of TFs specificity and function across diverse eukaryotic groups. While some features have been well-conserved during the eukaryotic radiation such as binding sites of MADS proteins, others have varied through the emergence of new binding sites or structures such as the *P. infestans* bZIPs containing Cys in their DNA-binding domain and HSF factors lacking canonical oligomerization regions. Our data will also help connect *P. infestans* TFs with their genic targets, providing insight into the regulation of transcription and life-stage transitions. This will help reveal the molecular components of processes such as sporulation, germination, and host colonization that are central to plant disease and potentially defeatable by targeted inhibitors.

### Electronic supplementary material

Below is the link to the electronic supplementary material.

Supplementary Material 1

Supplementary Material 2

Supplementary Material 3

## Data Availability

The data generated or analyzed during this study are included in the supplementary files or in NCBI under Bioproject PRJNA1069773.

## Abbreviations

bHLH: basic Helix-Loop-Helix
bZIP: basic Leucine Zipper Domain
ChIP-seq: Chromatin Immunoprecipitation sequencing
RM ANOVA: Repeated measures analysis of variance
CPM: Counts Per Million
CRM: *Cis*-Regulatory Module
EMSA: Electrophoretic Mobility Shift Assay
FPKM: Fragments Per Kilobase of Transcript Per Million Mapped Reads
HSF: Heat Shock Factor
HTH: Helix-Turn-Helix
PBM: Protein-Binding Microarray
TF: Transcription Factor
TMM: Trimmed Mean of M values
