# Supplementary material for: Transcription factor binding specificities of the oomycete Phytophthora infestans reflect conserved and divergent evolutionary patterns and predict function
Source: BMC Genomics. 2024 Jul 23;25:710. doi: 10.1186/s12864-024-10630-6 (PMC11267843; doi:10.1186/s12864-024-10630-6)
Supplement: Supplementary file 3 — Supplementary Material 3 [file 12864_2024_10630_MOESM3_ESM.pdf]

Fig. S1. Phylogenetic trees of the seven major transcription factor families, based on the amino acid sequences of their DNA-binding domains. Five-digit names represent proteins from *P. infestans* (trimmed of their "PITG" prefix), with asterisks indicating those that yielded a binding preference in the PBMs. Also included are the human (Hs prefix) and *A. thaliana* (At prefix) TFs referred to in the text. Bootstrap values above 50% are shown at the nodes.

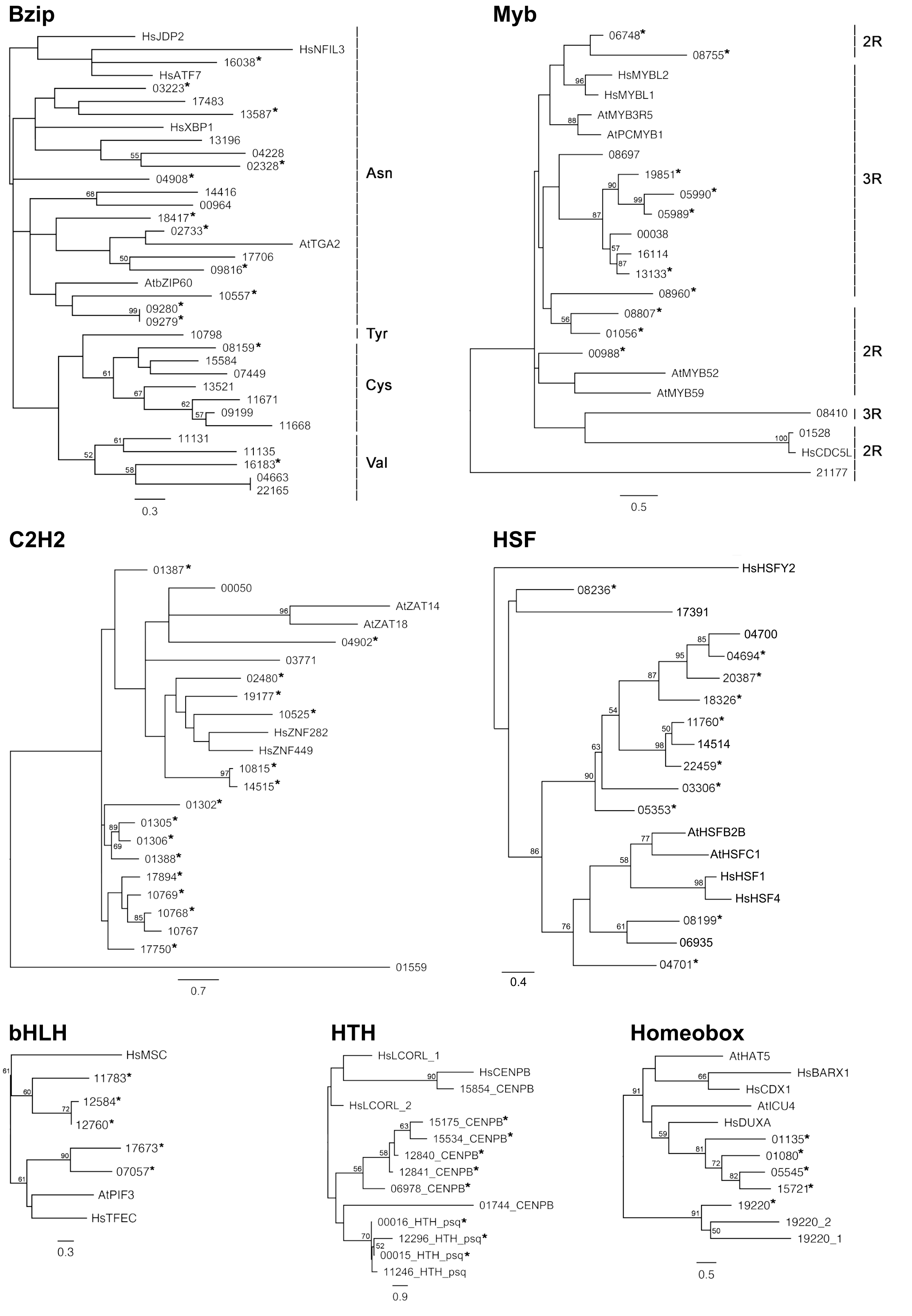

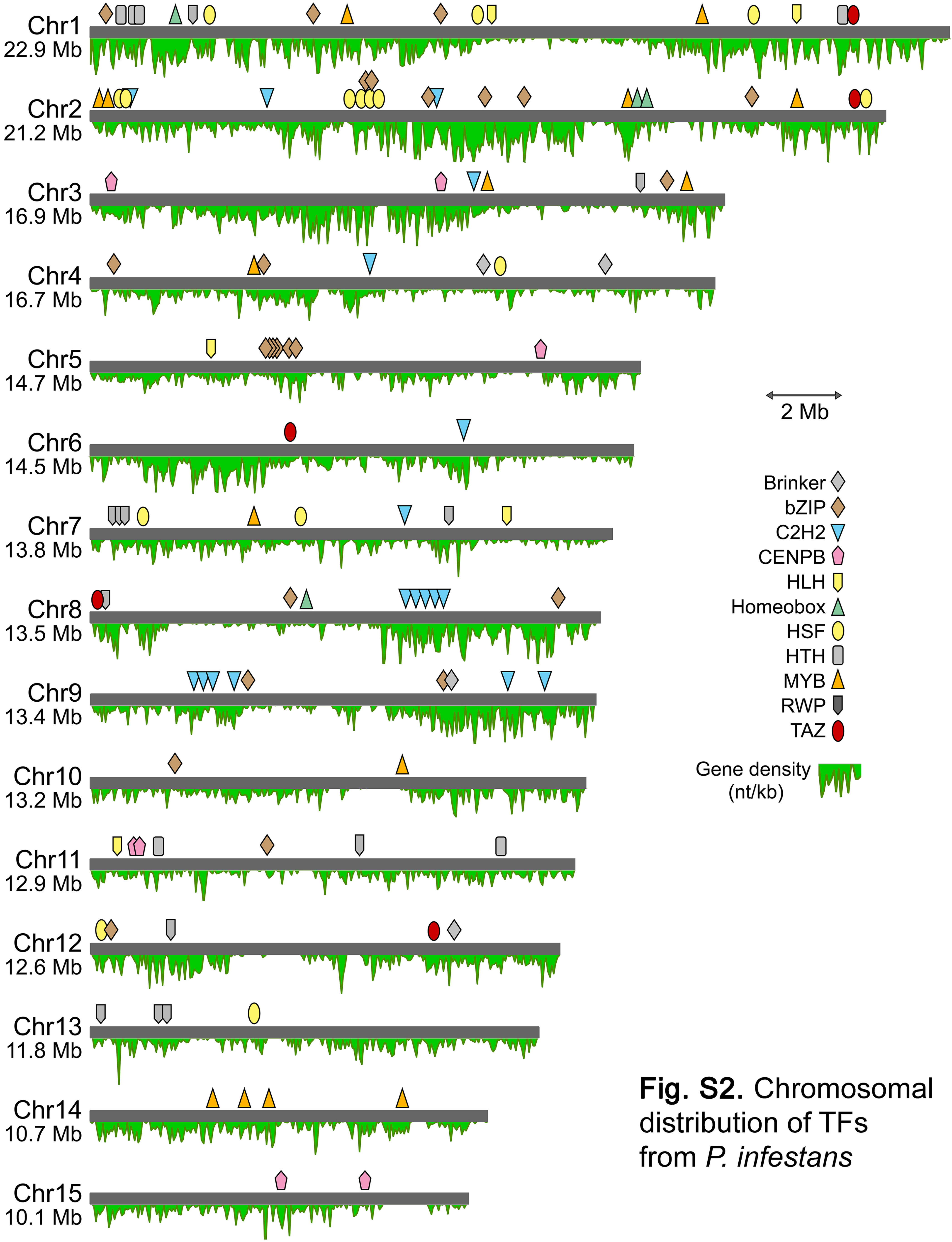

Fig. S2. Chromosomal distribution of TFs from *P. infestans*

FIG S3. Alignments of top twenty eight-mers extracted from the PBMs.

```
# PITG_06416_E2F_TDP
---CGCGCGCG-
GCGCGCGC----
---CGCGGGAA-
---GGCGGGAA-
----GCGGGAAA
--TGGCGCCA--
--TGGCGCCA--
---GGCGCCAA-
---CGCGCCAT-
---CCCGCCAT-
---CCCGCCAC-
--TCCCGCCA--
--TCCCGCCA--
---CCCGCCAA-
-TTCCCGCC---
TTTCCCGC----
-TTCCCGCG---
---CGCGCCAA-
---CGCGCGAA-
----GCGCGAAA
```

```
# PITG_12840_CENP-B_N
-GCTTAACA-
-GCTTAACA-
-GTTTAACA-
-GGTTAACA-
--GTTAACAC
--GTTAACAA
--GTTAACAA
-TGTTAACA-
-TGTTAACA-
-AGTTAACA-
-AGTTAACA-
--GTTAACAG
--GTTAACAG
--GTTAACAT
--GTTAACAT
CAGTTAAC--
AAGTTAAC--
GAGTTAAC--
-AGTTAACT-
-AGTTAACT-
```

```
# PITG_12841_CENP-B_N
--GTTAAACA--
--TGTTAACG--
--TGTTAACT--
-ATGTTAAC---
---GTTAACAG-
--AGTTAACA--
--GGTTAACA--
--TGTTAACA--
--TGTTAACA--
---GTTAACAT-
--AGTTAACT--
-CAGTTAAC---
-CTGTTAAC---
-TTGTTAAC---
-GTGTTAAC---
CGTGTTAA----
-GCGTTAAC---
----TTAACACG
--GTTTAACA--
--TGTTTAAC--
```

```
# PITG_07059_MADs
TAAATATA-
TAAATATA-
-AAATATAG
CTAAAATA-
TAAAAATA-
TAAAAATA-
-AAAAATAG
CAAAAATA-
CAAAAATA-
TATATATA-
TATATATA-
-ATATATAT
TATAATTA-
TAATAATA-
TAATAATA-
TATTAATA-
TATTAATA-
TATAAATA-
TATAAATA-
-ATAAATAG
```

```
# PITG_06978_CENP-B_N
--CGAACCTA-
---GAACTTAG
-CCGAACTT--
-GCGAACTT--
--CGAACTTA-
--CGAACTTA-
---GAACTTAA
--CGTACCTA-
--CGCACTTA-
--CGCACTTA-
--CGTACTTT-
-GCGTACTT--
-GCGTACTT--
--CGTACTTA-
--CGTACTTA-
TGCGTACT---
---GTACTTAA
---GTACTTAA
--TGTACTTA-
---GTACTTAG
```

```
# PITG_00215_CG-1
--GGTTTTAT
--GGTTTTAT
--TGGTTGCA
-GGGGTGGG-
GGGGGTGG--
-GGGGTGGC-
-GGGGTGGC-
TGGGGTGG--
TGGGGTGG--
-GGGGTGGA-
-GGGGTGGA-
--GGGTGGCC
--GGGTGGAG
TTGGGTGG--
--GGGTGGGG
--GGATGGAG
--GGATGGCC
GGGGATGG--
TGGCCTGG--
-GGCATGGC-
```

```
# PITG_15175_CENP-B_N
-GCTTAACG-
-GCTTAACA-
-GCTTAACA-
GGCTTAAC--
CGGTTAAC--
AGGTTAAC--
GGGTTAAC--
-GGTTAACA-
-GGTTAACA-
--GTTAACAA
-TGTTAACA-
-GGTTTACA-
-GGTTTACA-
AGGTTTAC--
-GATTTACA-
-GATTAACA-
-GATTAACA-
-GTTTAACA-
-GTTTAACA-
CGTTTAAC--
```

```
# PITG_15534_CENP-B_N
--TTTAACAC
--TTTAACAC
--TTTAACAG
-TTTTAACA-
-TTTTAACA-
--TTTAACAA
--TTTAACAA
-ATTTAACA-
-ATTTAACA-
--TTTAACAT
--TTTAACAT
-GTTTAACA-
-GTTTAACA-
TGTTTAAC--
TGTTTAAC--
-GTTTAACG-
-GTTTAACG-
GGTTTAAC--
-GTTTAACT-
CGTTTAAC--
```

```
# PITG_11783_HLH
-GCAACTGC-
--CAGCTGCT
-CCAGCTGC-
--CAGCTGCC
--CAGCTGCC
-ACAGCTGC-
-ACAGCTGC-
--CAGCTGCG
--CAGCTGCG
--CAGCTGCA
--CAGCTGCA
-GCAGCTGC-
-GCAGCTGC-
AGCAGCTG--
-GCAGCTGA-
--CAGCTGAC
--CAGCTGAC
-TCAGCTGA-
--CAGCTGTC
ATCAGCTG--
```

```
# PITG_17673_HLH
---ATATCACA
--TACACACA-
--AACGTTCT-
---ACGTTCCT
-GAACGTTC--
--CACGTGTC-
---ACGTGTCC
AGCACGTG---
--CACGTGCC-
--CACGTGCC-
--CACGTGCT-
---ACGTGCCA
-GAACGTGC--
AGAACGTG---
---ACGTGCTT
-GCACGTGC--
-GCACGTGC--
--CACGTGCA-
-ACACGTGC--
-ACACGTGC--
```

```
# PITG_12760_HLH
AACATCTG--
AACAGCTG--
AACAGCTG--
-ACAGCTGT-
-ACAGATGG-
-ACAGATGG-
AACAGATG--
-CCAGATGG-
--CAGATGGT
-CCATATGG-
-CCATATGG-
--CATATGGT
--CATATGGT
--CATATGGC
-ACATATGG-
-ACATATGG-
AACATATG--
AACATATG--
GACATATG--
-ACATATGT-
```

```
# PITG_12584_HLH
AACAGCTG--
AACAGCTG--
AACAGATG--
-ACAGATGG-
-ACAGATGG-
-CCAGATGG-
--CATATGGA
--CATATGGA
--CATATGGC
-ACATATGG-
-ACATATGG-
-CCATATGG-
-CCATATGG-
--CATATGGT
--CATATGGT
AACATATG--
AACATATG--
GACATATG--
-ACATATGT-
-ACATATGT-
```

```
# PITG_07057_HLH
-ACACGCGC-
-GCACGCGC-
-GCACGCGC-
--CACGCGCC
-ACACGCGT-
-ACACGCGT-
GACACGCG--
--CGCGTGCC
-ACGCGTGC-
--CGCGTGTC
--CACGCGTG
-GCACGTGC-
-GCACGTGC-
--CACGTGCC
--CACGTGCC
-ACACGTGC-
-ACACGTGC-
--CACGTGTC
-ACACGTGT-
GACACGTG--
```

```
# PITG_23160_AP2
-TAAATGTA
-ACAAAATA
ATACAAAT-
-TTTATAAT
TTATATAA-
-TAAATAAT
-ATAGTAAT
-AAAATAAA
-ATAATAAA
AATAATAA-
-ATAATAAT
-TAATAATA
-TTAAAGTA
-AACATTTA
-AATAAATT
-AATATATT
-AATATATT
AAATATAT-
-TATAAATC
-TATAAATA
```

```
# PITG_10442_CSD
-GCAATTGC-
GGCAGCTG--
GGCAGCTG--
-GCAGCTGA-
-GCAGCTGA-
TGCAGCTG--
TGCAGCTG--
AGCAGCTG--
AGCAGCTG--
--CAGCTGAC
--CAGCTGAC
-TCAGCTGA-
-TCAGCTGA-
-GCAGCTGT-
-GCAGCTGT-
-GCAGCTGG-
-GCAGCTGC-
-GCAGCTGC-
CGCAGCTG--
CGCAGCTG--
```

```
# PITG_11760_HSF_DNA-bind
GTTCCATA---
-TTCCATAT--
--TTCGAAAA-
--TTCGAAAT-
TTTTCGAA---
ATTTCGAA---
CTTTCGAA---
ACTTCGAA---
-TTTCGAAT--
-TTTCGAAA--
-TTTCGAAA--
-GTTCGAAA--
-ATTCGAAT--
TATTCGAA---
AATTCGAA---
AATTCGAA---
-ATTCGAAA--
AAATAGAA---
-TATGGAAT--
---TGGAACAT
```

```
# PITG_04701_HSF_DNA-bind
-----TTCCAGAA-
-----TTCCAGAA-
-----TTCGAGAA-
-----TTCGAGAA-
-AATGTTCT-----
----CTTCTAGA--
----GTTCTAGA--
----GTTCTAGA--
----ATTCTAGA--
----ATTCTAGA--
-----TTCTAGAA-
-----TTCTAGAA-
------TCTAGAAA
-----ATCTAGAA-
-----ATCTAGAA-
GAACATTC------
--GTGTTCGA----
----ATTTCGAA--
---CATTCGAA---
----ATTCGAAT--
```

```
# PITG_20387_HSF_DNA-bind
---TCCTTCGA---
----TCTTCCAC--
---TTCTTCCA---
---ATGTTCCA---
---TATTTCCA---
---ACTTTCTA---
----TTTTCTAT--
------TTCTATTT
---ATTTTCCA---
AAAATTTT------
---TTTTTCCA---
----TTTTCCAT--
------TTCCACAA
-----TTTCGAAA-
-----CTTCCATA-
------TTCCATAT
GAAATTTC------
-----TTTCCATA-
-----TTTCCATA-
------TTCCATAA
```

```
# PITG_18326_HSF_DNA-bind
GAAATTTC-----
AATTTTCT-----
--TTTTCTAC---
--TTTTCTAT---
-TTTTTCTA----
TTTCTTCC-----
-TCTTTCCA----
-TTTTTCCA----
--TTTTCCAC---
--TTTTCCAC---
--TTTTCCAT---
--TTTTCCAT---
-ATTTTCCA----
---TTTCCATG--
---TTTCCATA--
---TTTCCACA--
-----TCCATGGA
-----TCCACGGA
-TATTTCCA----
---TTTCCACG--
```

```
# PITG_08236_HSF_DNA-bind
-GAGAAAAT-
-GAGAATAT-
-GAGAATAC-
-GAGAACAA-
-GAGAACAC-
-GAGAACAC-
CGAGAACA--
AGAGAACA--
GGAGAACA--
-GAGAACAT-
GAAGAATT--
AGAGAATT--
--AGAATTCT
--AGAATTCT
-GAGAATTC-
GAAGAATA--
AAAGAACA--
GAAGAACA--
GAAGAACA--
-AAGAACAA-
```

```
# PITG_08199_HSF_DNA-bind
GAACATTC--
-AGAATTCT-
-AGAATTCT-
AAGAATTC--
AAGAATTC--
-CGAATTCG-
CCGAATTC--
GAGAATTC--
--GAATTCCG
--GAATTCCA
-GGAATTCC-
-GGAATTCC-
--GAATTCGC
-AGAATTCG-
--GAATTCCT
--GAATTCCT
TAGAATTC--
TAGAATTC--
-AGAATTCC-
-AGAATTCC-
```

```
# PITG_04694_HSF_DNA-bind
--TTTCGAAA--
--TTTCGAAA--
-TTTTCGAA---
---GTGGAAAG-
-TTATGGAA---
-TTATGGAA---
-ATATGGAA---
--TATGGAAA--
--TATGGAAA--
AATATGGA----
-AAATGGAA---
TCCATGGA----
----TGGAAAAT
-TTATAGAA---
TTTATAGA----
---ATAGAAAG-
----TAGAAGAG
-TTCTAGAA---
--TCTAGAAA--
----TAGAAAGT
```

```
# PITG_03306_HSF_DNA-bind
-GAAAAGAA-
--AAAAGAAT
--TTCCGGAA
-ATTCCGTA-
--TTCCGAAT
--TTCCGAAT
-ATTCCGAA-
-ATTCCGAA-
TATTCCGA--
GGTTCCGA--
--TTCCGAAA
--TTCCGAAA
-GTTCCGAA-
-GTTCCGAA-
--TTCTTTTT
--TTCTTTTT
GGTTCTTT--
GGTTCTTT--
-GTTCTTTT-
-GTTCTTTT-
```

```
# PITG_22459_HSF_DNA-bind
TTATGGAA----
AAAAAGAA----
ATT----ATTCC
----AGAATTCT
ATATAGAA----
ATTTAGAA----
--TTCGATTT--
TGTTCGAA----
-TTTCGAAA---
-TTTCGAAA---
-TTTCGAAC---
-TTTCGAAC---
-TTTCGAAT---
TTTTCGAA----
TTTTCGAA----
ATTTCGAA----
ATTTCGAA----
TATTCGAA----
AATTCGAA----
--TTCGAATA--
```

```
# PITG_05353_HSF_DNA-bind
--AAATAGAA---
--TTTTAGAA---
---TTCTAAAA--
---GTTCGAAA--
---TTTCGAAA--
---TTTCGAAA--
---ATTCGAAA--
----TTCGAAAT-
----TTCGAAAA-
-----TCGAAAAA
--ATTTCGAA---
--TTTTCGAA---
-AATTTCGA----
--GTTTCGAA---
---TTTCGAAC--
AAATTTCG-----
----TTCGAATA-
---ATTCGAAT--
--AATTCGAA---
--AATTCGAA---
```

```
# PITG_00015_HTH_psq
TTGTAACA-
-TGTAACAA
-TGTAACAG
GTGTAACA-
GTGTAACA-
-TGTAACAT
-CGTTACAC
GCGTAACA-
-CGTAACAT
TCGTAACA-
TCGTAACA-
-CGTAACAC
-CGTAACAC
ACGTAACA-
-CGTAACAA
-CGTAACAA
CCGTAACA-
CCGTAACA-
-CGTAACAG
-CGTAACAG
```

```
# PITG_00016_HTH_psq
-TGGAACAG
TTGTAACA-
-TGTAACAA
GTGTAACA-
-TGTAACAT
-CGTTACAG
-CGTTACAC
TCGTAACA-
TCGTAACA-
GCGTAACA-
GCGTAACA-
-CGTAACAC
-CGTAACAC
ACGTAACA-
-CGTAACAA
-CGTAACAA
CCGTAACA-
CCGTAACA-
-CGTAACAG
-CGTAACAG
```

PITG_12296_HTH_psq
GCGTAACA--
GCGTAACA--
-CGTAACAT-
ACGTAACA--
-CGTAACAC-
-CGTAACAC-
TCGTAACA--
TCGTAACA--
-CGTAACAA-
-CGTAACAA-
-CGTAACAG-
-CGTAACAG-
CCGTAACA--
CCGTAACA--
TTGTAACA--
-TGTAACAA-
GTGTAACA--
GTGTAACA--
-TGTAACAG-
--GTAACAGT

PITG_08159_bZIP_1
-CGATAACA-
--GTGATCAC
-AGTTATCA-
-AGTTATCA-
GTGTTATC--
-TGTTATCA-
--GTTATCAC
--GTTATCAC
--GTTATCAG
--GTTATCAT
--GTTATCAA
--GTTATCAA
-CGTTATCG-
-CGTTATCA-
-CGTTATCA-
CCGTTATC--
CCGTTATC--
-CGTTATCC-
GCGTTATC--
GCGTTATC--

PITG_09816_bZIP_1
--ATTATATA-
--CATATATG-
---ATATATGA
---ATATACAT
-GAATATAT--
--AATATATC-
-AAATATAT--
-AAATATAT--
--AATATATT-
--AATATATT-
CAAATATA---
ATAATATA---
---ATATATAT
CTAATATA---
-GAATATTC--
-AAATATTC--
--AATATTCG-
--AATATTTC-
-AAATATTA--
-ACAAATAT--

PITG_18417_bZIP_1
-TACGTCAC--
-TACGTCAC--
-TGCGTCAT--
-TGCGTCAT--
---CGTCATCG
--GCGTCATC-
--GCGTCATC-
--ACGTCATT-
--ACGTCATA-
-CACGTCAT--
-TACGTCAT--
-TACGTCAT--
-CGCGTCAT--
TGACGTCA---
TGACGTCA---
-GACGTCAT--
-GACGTCAT--
---CGTCATCA
--ACGTCATC-
--ACGTCATC-

PITG_02328_bZIP_1
-GTAATTAC-
-GTAATTAC-
CGTAATTA--
--TAATGACT
-TTAATGAC-
--TAATGACA
-ATAATGAC-
-ATAATGAC-
-GTAATGAC-
-GTAATGAC-
--TAATGACG
--TAATGACG
-ATAATGAT-
--TAATGATT
--TAATGATT
--TAATGATG
--TAATGATG
-GTAATGAT-
-GTAATGAT-
TGTAATGA--

PITG_03223_bZIP_1
-ATGTAACA--
--TGTAACAT-
--TGTAACAG-
-GTGTAACA--
-ATGTAACG--
-ATGTAACG--
---GTAACGTT
---GTAACGTA
TGTGTAAC---
-GTGTAACG--
--AGTAATGT-
--TGTAATGT-
--TGTAATGA-
-GTGTAATG--
-TTGTAATG--
--TGTAATGC-
--TGTAATGC-
TGTGTAAT---
-TGGTAATG--
---GTAATGGT

PITG_16183_bZIP_1
---CATCGATG
--AAACGTTT-
--GCACATAT-
---AACATGTT
--ATCATGAT-
TAGCAATC---
--GTAATCAA-
ACGTAATC---
-CGTAATCA--
-TGCAATCA--
-CGCAATCA--
--TTGCGCAA-
---CGCGCGCG
--TCGCGCGA-
--ATGTGTAA-
--ACATACAC-
-CGTATACG--
--GTGATCAC-
--GTGTACAC-
-TGTGCACA--

PITG_04908_bZIP_1
TTGACTCA-
-TGACTCAA
-TGACTCAG
-TGACTCAG
CTGACTCA-
CTGACTCA-
-TGACTCAT
-TGACTCAT
GTGACTCA-
GTGACTCA-
ATGACTCA-
ATGACTCA-
-TGACTCAC
-TGACTCAC
-TGACGTCA
-CGATGACG
ATGACGCA-
-TGACGCAT
-TATATTAT
-TTTTGTAT

PITG_13587_bZIP_1
-CAATGATG
ACATGTTA-
-CAATTGTT
ACACGTGT-
-CACGTGTA
-CAGTTACA
-TATTTGCA
GTACAATA-
-TGTTAACA
-TGTTAACA
TTGTTAAC-
ATGTACAT-
GTGTACAC-
-TGTGCACA
TTGTAATA-
ATGTGAAG-
ACGTGAAA-
-TGTGAAAT
-CGAATTCG
GAAATTTC-

```
# PITG_09279_bZIP_1
-TGACGTCA--
---ACGTGTCC
TACACGTG---
--CACGTGTC-
--CACGTGTC-
-ACACGTGT--
-ACACGTGT--
-ACACGTGG--
-ACACGTGG--
--CACGTGGC-
--CACGTGGC-
-CCACGTGG--
-CCACGTGG--
--TACGTGGC-
--TACGTGGC-
---ACGTGGCA
---ACGTGGCA
---ACGTGGCG
-TGACGTGG--
---ACGTGGCC
```

```
# PITG_02733_bZIP_1
TGACGTCA--
TGACGTCA--
-GACGTCAT-
-GACGTCAT-
-GACGTCAC-
-GACGTCAC-
-TACGTCAC-
-TACGTCAC-
--ACGTCACG
--ACGTCACG
--ACGTCACT
--ACGTCACT
--ACGTCACC
-CACGTCAC-
-CACGTCAC-
CCACGTCA--
-CACGTCAT-
-CACGTCAT-
--ACGTCATC
--ACGTCATC
```

```
# PITG_09280_bZIP_1
--ACACGTGG--
--ACACGTGG--
--CCACGTGG--
--CCACGTGG--
---CACGTGGC-
---CACGTGGC-
--ACACGTGT--
--ACACGTGT--
---CACGTGTC-
---CACGTGTC-
---TACGTGGC-
---TACGTGGC-
----ACGTGGCG
----ACGTGTCC
----ACGTGTCA
GATGACGT----
--TGACGTGG--
--TGACGTGG--
----ACGTGGCA
----ACGTGGCA
```

```
# PITG_16038_bZIP_1
TTACGTAA----
-TACGTAAT---
-TTAGTAAT---
---TGTAATAA-
----GTAATAAT
--ATGTAATA--
---TGTAATAT-
--ATGTAATC--
---TGTAATCG-
-TATGTAAT---
-TGTGTAAT---
-TGTGTAAT---
---TGTAATGC-
---TGTAATGT-
-TCTGTAAT---
----GTAATTAC
----GTAATTAC
---TGTAATTA-
-ACTGTAAT---
--CTGTAATT--
```

```
# PITG_10557_bZIP_1
--GTTGTGGT
-TGTTGGTC-
--GCACGAAC
--GATCGATC
--GTTCGTGT
-TGTTCGTG-
-TGTTCGAG-
ATGTTCGA--
-TGTTCGAT-
-TGTTCGAC-
-TGTTCGAC-
--GTTCGAGC
--GTTCGAAC
--GTTCGAAC
-TGTTCGAA-
-TGTTCGAA-
GTGTTCGA--
GTGTTCGA--
-TGTGTGTA-
--GTTTGTTG
```

```
# PITG_01305_zf-C2H2
-GATATGTT--
--TTTTGCAC-
---TGTGCACA
---TGTGCACA
---GATGCATC
--AGATGCAC-
--TCGTGCAC-
-ATCGTGCA--
GCACGTGC---
---CGTGCACG
--ACGTGCAC-
-GCAATGCA--
TGCAGTGC---
-ACAGTGCA--
GGATGTGC---
-GATGTGCA--
--GTGTGCAC-
-TGTGTGCA--
--CTGTGCAT-
--CTGTGCAC-
```

```
# PITG_10815_zf-C2H2
--CCCATCAC-
--CCCATCAC-
-GCCCATCA--
AGCCCATC---
-ACCCATCC--
--CCCATCCT-
-GCCCATCC--
-GCCCATCC--
--CCCATCCC-
--CCCATCCC-
TGCCCATC---
TGCCCATC---
--CCCATCTC-
--CCCATCTC-
---CCATCTCC
-GCCCATCT--
CGCCCATC---
--CTCATCCC-
TGCGCATC---
--CGCATCCC-
```

```
# PITG_01306_zf-C2H2
---AGTGGGTG---
---TAAACACA---
------GCAGGAAG
-----TGCATTCT-
---AGTGCACA---
----GTGCACAT--
----GTGCACAA--
---GGTGCACA---
----GTGCACAC--
---TGTGCACA---
---TGTGCACA---
--CAGTGCAC----
-GTGGTGCA-----
--CCGTGCAC----
---CGTGCACG---
---CGTGCACG---
----GTGCACGA--
-GATGTGCA-----
GGATGTGC------
----GTGCACGG--
```

```
# PITG_17750_zf-C2H2
--CCCATCCT
--CCCATCTT
--CCCATCTT
--CAAATCGC
-GCAAATCT-
AGCAAATC--
-GCAAATCC-
-GCAAATCC-
--CAAATCCT
--CAAATCAT
-GCAAATCA-
-GCAAATCA-
TGCAAATC--
TGCAAATC--
TGCACATC--
TGCACATC--
-GCACATCC-
-GCACATCC-
TGCAGATC--
-GCAGATCT-
```

```
# PITG_10768_zf-C2H2
--GATTTGTA
-CGATTTGC-
-TGATTTGC-
--GATTTGCA
--GATTTGCA
--GATTTGCG
--GATTTGCG
--GATTTGCC
--GATTTGCC
--GATTTGCT
-AGATTTGC-
-AGATTTGC-
ATGATTTG--
AAGATTTG--
-GGATTTGC-
-GGATTTGC-
--GATTTGAA
AGGATTTG--
AGGATTTG--
-GGATTTGA-
```

```
# PITG_10769_zf-C2H2
--CCCATCTT
--CCCATCTT
--CCCATCCC
--CCCATCCC
--CCCATCCT
--CCCATCCT
-TCCCATCC-
-TCCCATCC-
-GCAGATCT-
TGCACATC--
TGCACATC--
-GCACATCC-
-GCACATCC-
--CAAATCCC
TGCAAATC--
TGCAAATC--
--CAAATCCT
--CAAATCCT
-GCAAATCC-
-GCAAATCC-
```

```
# PITG_01387_zf-C2H2
---AATATATT
-GGAATTCC--
CGGGAATC---
-ATGATCAT--
--GAATCCGA-
--GAATCCTA-
ATGGATTC---
-TGGATCCA--
ATGGATCC---
-CGGATCCG--
-CGGATCCG--
---GATCCGGA
CGGGATCC---
-GGGATCCC--
AGGGATCC---
-AGGATCCT--
-AGGATCCT--
--GGATCCTA-
---GATGCATC
--AAATGCAT-
```

```
# PITG_10525_zf-C2H2
--CCTCCGGA-
---CTCCGGAT
--CCTTCGGA-
--CCTTCGGA-
---CTTCGGAT
---CTTCGGAT
--CCGTCGGA-
--CCGTCGGA-
---CGTCGGAT
CGCCGTCG---
CGCCGTCG---
-GCCGTCGG--
---CGTCGGAG
--ACGTCGGA-
--ACGTCGGA-
CGTCGTCG---
--TCGTCGGA-
--TCGTCGGA-
---CGTCGGAA
---CGTCGGAA
```

```
# PITG_04902_zf-C2H2
--AAGTACTT
TAACGAAA--
-TGTATACA-
-AAAAAATA-
-ATTATATA-
-TATTAACA-
-TATTAATA-
-TATTAATA-
ATATTAAT--
--AAAAATAA
-TTTAAATT-
-AATAAATT-
--ATAAATTA
--ATACATTA
--ATATAATA
-ATAATATA-
-TATATATA-
--ATATATAT
-AATATATT-
--ATATATTA
```

```
# PITG_19177_zf-C2H2
-AGAGTGAT-
-AGAGTGAT-
CAGAGTGA--
CAGAGTGA--
-AGAGTGAC-
GAGAGTGA--
GAGAGTGA--
-AGAGTGAA-
AAGAGTGA--
AAGAGTGA--
-TGAGTGAT-
-TGAGTGAT-
GTGAGTGA--
GTGAGTGA--
-TGAGTGAA-
ATGAGTGA--
--GAGTGATT
AGGAGTGA--
--GAGTGATG
--GAGTGATG
```

```
# PITG_01388_zf-C2H2
-AAATGCAT----
-TATTGATC----
AATATGAT-----
---ATGATCAT--
---ATGATCAT--
--TATGATCA---
-AAATGATT----
--AATGATTC---
-GAATGATT----
--AATGATCC---
--AATGATCC---
AAAATGAT-----
AAAATGAT-----
-AAATGATC----
-AAATGATA----
-AAATGATA----
--CGTGCACG---
-----GTTCAAAC
---GTGCAAAA--
--TGTGCACA---
```

```
# PITG_17894_zf-C2H2
----AGATAGGA
---TGTGCACA-
---CGTGCACG-
----GTGCACGA
--TGGGATAA--
---GGGATAAC-
---AGGATAAT-
---AGGATAAG-
--TAGGATAA--
--TAGGATAA--
--CAGGATAA--
---AGGATAAC-
--GAGGATAA--
--GAGGATAA--
---AGGATCCT-
---AAGATCTT-
ATCCGGAT----
---ATGATCAT-
---ATGATCAT-
---ATCATGAT-
```

```
# PITG_14515_zf-C2H2
--CTCATCCC-
---CCATCTCC
--CCCATCTC-
-GCCCATCT--
-GCCCATCT--
CGCCCATC---
CGCCCATC---
-ACCCATCC--
--CCCATCCT-
--CCCATCCT-
-GCCCATCC--
-GCCCATCC--
--CCCATCCC-
--CCCATCCC-
TGCCCATC---
--CCCATCAC-
--CCCATCAC-
-GCCCATCA--
AGCCCATC---
AGCCCATC---
```

PITG_01302_zf-C2H2

```
-CGTGCACA
-TGTGCACA
-TGTGCACA
GTGCACAC-
-TGCATGCA
CCGTGCAC-
ACGTGCAC-
-CGTGCACG
-CGTGCACG
-TCCGTGCA
GTGCACGA-
GTGCACGG-
-TGCACGAT
-TGTGCACG
GTGCACAT-
GTGCACAG-
-GGTGCACC
GCACGTGC-
CGGTGCAC-
CGGTGCAC-
```

PITG_02480_zf-C2H2

```
--GCACGTGC-
-CTCACGTC--
--TCACGTCG-
--TCACGTCC-
-TTCACGTC--
-TTCACGTC--
--TCACGTCA-
--TCACGTCA-
---CACGTCAA
---CACGTCAA
--GCACGTCA-
--GCACGTCA-
-TGCACGTC--
-TGCACGTC--
---CACGTCGA
---CACGTCAC
---CACGTCAT
--TCACGTGA-
--TCACGTGA-
TTTCACGT---
```

PITG_01080_Homeobox

```
-CCCATCAT--
-CCCATCAC--
-CCCATCAC--
GCCCATCA---
GCCCATCA---
ACCCATCA---
ACCCATCA---
-CCCATCAA--
-CCCATCAA--
--CCATCAAT-
--CCATCAAT-
---CATCAATC
---CATCAATC
--CCATCATA-
AGCCATCA---
AGCCATCA---
-GCCATCAA--
-GCCATCAA--
--CCATCAAA-
--CCATCAAA-
```

PITG_01135_Homeobox

```
--TGGTCAAT
GCTAGTCA--
ACTAGTCA--
-CTAGTCAA-
-CTAGTCAA-
--TAGTCAAA
--TAGTCAAA
--TAGTCAAT
CGTAGTCA--
GGTAGTCA--
-GTAGTCAC-
-GTAGTCAT-
-GTAGTCAT-
AGTAGTCA--
AGTAGTCA--
TGTAGTCA--
TGTAGTCA--
-GTAGTCAA-
-GTAGTCAA-
--TAGTCAAC
```

PITG_05545_Homeobox

```
TGACGTCA---
TGACGTCA---
--GTGACAGC-
--GTGACAGC-
ACATGTCA---
-CATGTCAC--
--GTGTCAAC-
ATGTGTCA---
AGCTGTCA---
-CCTGTCAA--
ATCTGTCA---
---TGTCAACG
-CGTGTCAA--
-CGTGTCAA--
GCGTGTCA---
GCGTGTCA---
TCGTGTCA---
TCGTGTCA---
ACGTGTCA---
-CGTGTCAC--
```

PITG_15721_Homeobox

```
TGACAAGC--
-GCGCATCA-
-GCCTATCA-
-ATCAATCA-
-GTCAATCA-
-GCCAATCA-
-GCCAATCA-
--CCAATCAA
-GCTAATCA-
-GCTAATCA-
--CAAATCAT
-GCAAATCA-
-GGTAGTCA-
-GCTAGTCA-
-GGTTATCA-
-GCTTATCA-
-GCTTGTCA-
-GCGTGTCA-
-GCGTGTCA-
--CGTGTCAA
```

PITG_19220_Homeobox

```
CATATATG---
CATATATG---
--TTAATATT-
--TATATGGA-
--TATATGCA-
---AAATGCAT
-CATAATGA--
--ATAATGCA-
--GATATGTA-
---TAATGTAT
---TAATGTAC
--ATAATGTA-
--ATAATGTA-
-AATAATGT--
-CATAATGT--
-CATAATGT--
---ATGTACAT
CATTAATG---
CATTAATG---
-ATTAATGA--
```

PITG_17861_BrkDBD

```
-GGCGCCAT--
-CCCGCCAC--
-TCCGCCAC--
ATCCGCCA---
ATCCGCCA---
--CCGCCATT-
-TCCGCCAT--
--CCGCCATC-
--CCGCCATA-
--CCGCCATA-
-ACCGCCAT--
-ACCGCCAT--
ACCCGCCA---
--CCGCCATG-
--CCGCCATG-
-CCCGCCAT--
-ACCGCCAC--
---CGCCACCC
--CCGCCACC-
--CCGCCACC-
```

PITG_19429_BrkDBD

```
GGGGGGTA--
TACTAATT--
ATTTAGTA--
-AATTATAC-
AATTAGTG--
-AATAGTGT-
TATTTGTA--
TAATTGTA--
TAATTGTA--
TAATAGTA--
TAATAGTA--
-AATAGTAC-
TATTAATA--
TATTAATA--
-ATTAATAC-
-AATAATAT-
TAATAATA--
ATATAATA--
TTATAATA--
--ATAATATA
```

PITG_08960_Myb_DNA-binding
AACCGGTC---
--CCGGTCGC-
-ACCGGTCG--
-ACCGGTCG--
TACCGGTC---
TACCGGTC---
--CCGGTCGT-
--CCGGTCGT-
CACCGGTC---
CACCGGTC---
--CCGGTCAT-
---CGGTCACG
--CCGGTCAC-
-ACCGGTCA--
-ACCGGTCA--
GACCGGTC---
GACCGGTC---
-ACCGGCCG--
-ACCGGCCG--
-ACCGGCCA--

PITG_06748_Myb_DNA-binding
GCAGTTAC--
ACAGTTAC--
ACAGTTAC--
-CAGTTACA-
-CAGTTACA-
-CGGTTTCA-
ACGGTTTC--
-CGGTTACA-
-CGGTTACA-
ACGGTTAC--
ACGGTTAC--
-CGGTTACG-
-CCGTTACA-
-CCGTTACA-
-CCGTTACT-
-CCGTTACG-
ACCGTTAC--
AACGTTAC--
GGCGTTAC--
--CGTTACAT

PITG_05990_Myb_DNA-binding
AAAAAATC---
--ATATCCAG-
-AATATCCA--
-AATATCCA--
GAATATCC---
-AGTATCCA--
-AGTATCCA--
---TATCCAGT
--AAATCCAG-
--AAATCCAG-
-AAAATCCA--
-AAAATCCA--
GAAAATCC---
AAAAATCC---
-AGAATCCA--
-AGAATCCA--
GAGAATCC---
--GAATCCAG-
---AATCCAGT
---AATCCAGT

PITG_13133_Myb_DNA-binding
-TATACGGA-
-TATACGGA-
ATATACGG--
ATATACGG--
-TATACGGT-
--ATACGGAG
--ATACGGAG
--ATACGGAT
AGATACGG--
--ATACGGTT
--ATACGGTT
AGATCCGG--
--ATCCGGAT
--ATCCGGAC
-TATCCGGA-
AGAGCCGG--
AGAGCCGG--
AGAGCCAG--
AGAGCCAG--
-GAGCCAGT-

PITG_05989_Myb_DNA-binding
ATTTAAAT--
-CTTGATAC-
-CTTGATTT-
ACTTGATT--
ACTTGATT--
-CTTGATTC-
-CTTGATTC-
-CTGGATAT-
ACTGGATA--
--TGAATTCA
--TGGATTCT
--TGGATTTA
ACTGGATT--
ACTGGATT--
-CTGGATTT-
-CTGGATTT-
--TGGATTCA
--TGGATTCA
-CTGGATTC-
-CTGGATTC-

PITG_00988_Myb_DNA-binding
--AACAGTTA--
--GGCAGTTA--
--AGCAGTTA--
--AACCGGTT--
---GCCGTTAC-
--AGCCGTTA--
---GCCGTTAT-
--GACCGTTA--
----CCGTTACA
---ACCGTTAC-
-AAACCGTT---
-CAACCGTT---
-CAACCGTT---
--AACCGTTT--
--AACCGTTT--
GTAACCGT----
-TAACCGTTC--
-TAACCGTT---
--AACCGTTA--

--AACCGTTA--

PITG_01056_Myb_DNA-binding
--CCGTTACT-
--CCGTTACG-
-ACCGTTAC--
-TCCGTTAC--
GGCCGTTA---
---CGTTACAT
--CCGTTACA-
--CCGTTACA-
-GCCGTTAC--
-GCCGTTAC--
--CGGTTTCA-
AACGGTTA---
-ACGGTTAC--
-ACGGTTAC--
--CGGTTACA-
--CGGTTACA-
-GCGGTTAC--
-GCGGTTAC--
GTCGGTTA---
--CGGTTACG-

PITG_08807_Myb_DNA-binding
--CCGTTTCA-
-TCCGTTTC--
--CGGTTACA-
--CGGTTACA-
--CCGTTACC-
--CCGTTACT-
--CCGTTACT-
--CCGTTACG-
--CCGTTACG-
-ACCGTTAC--
-ACCGTTAC--
-TCCGTTAC--
-TCCGTTAC--
GTCCGTTA---
GGCCGTTA---
---CGTTACAT
--CCGTTACA-
--CCGTTACA-
-GCCGTTAC--
-GCCGTTAC--

PITG_08755_Myb_DNA-binding
-CGGTTACG
-CGGTTACA
-CGGTTACA
-CAGTTACA
-CAGTTACG
-CAGTTACG
-CCGTTACT
-CCGTTACC
TCCGTTAC-
ACCGTTAC-
ACCGTTAC-
-CCGTTACG
-CCGTTACG
-CCGTTACA
-CCGTTACA
GCCGTTAC-
GCCGTTAC-
ACCGTTTC-
-CCGTTTCA
GCCGTTTC-

PITG_19851_Myb_DNA-binding

```
AACTGGAC--
-ACTGGACA-
--CTGACCAC
TACTGTAC--
-ACTGAACT-
-ACTGAACT-
-ACTGAACC-
-ACTGAACG-
GACTGAAC--
-ACTGAACA-
--CTGAACTC
-GCTGAACT-
--CTGAACTT
--CTGAACTT
AACTGAAC--
AACTGAAC--
TACTGAAC--
TACTGAAC--
TTCTGAAC--
--CTGAACAC
```

Fig. S4. Position-specific frequency matrices for 73 *P. infestans* transcription factors

MEME version 5

ALPHABET= ACGT

strands: + -

MOTIF PITG_03223_bZIP_1

letter-probability matrix: alength= 4 nsites= 10 E= 0
0.166667 0.166667 0.166667 0.500000
0.285714 0.071429 0.428571 0.214286
0.095238 0.047619 0.095238 0.761905
0.041667 0.041667 0.875000 0.041667
0.041667 0.041667 0.041667 0.875000
0.875000 0.041667 0.041667 0.041667
0.875000 0.041667 0.041667 0.041667
0.041667 0.458333 0.041667 0.458333
0.227273 0.045455 0.681818 0.045455
0.142857 0.214286 0.214286 0.428571
0.285714 0.142857 0.142857 0.428571

MOTIF PITG_12840_CENP-B_N

letter-probability matrix: alength= 4 nsites= 10 E= 0
0.285714 0.285714 0.285714 0.142857
0.470588 0.058824 0.294118 0.176471
0.041667 0.125000 0.750000 0.083333
0.041667 0.041667 0.041667 0.875000
0.041667 0.041667 0.041667 0.875000
0.875000 0.041667 0.041667 0.041667
0.875000 0.041667 0.041667 0.041667
0.041667 0.875000 0.041667 0.041667
0.761905 0.047619 0.047619 0.142857
0.272727 0.181818 0.272727 0.272727

MOTIF PITG_01302_zf-C2H2

letter-probability matrix: alength= 4 nsites= 10 E= 0
0.142857 0.285714 0.500000 0.071429
0.041667 0.291667 0.166667 0.500000
0.083333 0.083333 0.791667 0.041667
0.041667 0.416667 0.041667 0.500000

```
0.333333 0.041667 0.583333 0.041667
0.041667 0.750000 0.041667 0.166667
0.625000 0.041667 0.291667 0.041667
0.125000 0.666667 0.125000 0.083333
0.428571 0.142857 0.285714 0.142857


MOTIF PITG_09279_bZIP_1

letter-probability matrix: alength= 4 nsites= 10 E= 0
0.200000 0.200000 0.200000 0.400000
0.461538 0.230769 0.076923 0.230769
0.052632 0.631579 0.157895 0.157895
0.875000 0.041667 0.041667 0.041667
0.041667 0.875000 0.041667 0.041667
0.041667 0.041667 0.875000 0.041667
0.041667 0.041667 0.041667 0.875000
0.041667 0.083333 0.833333 0.041667
0.086957 0.043478 0.608696 0.260870
0.066667 0.800000 0.066667 0.066667
0.333333 0.333333 0.222222 0.111111


MOTIF PITG_04908_bZIP_1

letter-probability matrix: alength= 4 nsites= 10 E= 0
0.333333 0.250000 0.250000 0.166667
0.041667 0.083333 0.041667 0.833333
0.083333 0.041667 0.791667 0.083333
0.791667 0.041667 0.041667 0.125000
0.083333 0.750000 0.041667 0.125000
0.041667 0.041667 0.250000 0.666667
0.083333 0.708333 0.041667 0.166667
0.791667 0.125000 0.041667 0.041667
0.187500 0.187500 0.250000 0.375000


MOTIF PITG_08236_HSF_DNA-bind

letter-probability matrix: alength= 4 nsites= 10 E= 0
0.307692 0.153846 0.461538 0.076923
0.318182 0.045455 0.590909 0.045455
0.875000 0.041667 0.041667 0.041667
0.041667 0.041667 0.875000 0.041667
0.875000 0.041667 0.041667 0.041667
0.875000 0.041667 0.041667 0.041667
0.083333 0.500000 0.041667 0.375000
0.666667 0.041667 0.041667 0.250000
0.200000 0.466667 0.066667 0.266667
0.166667 0.166667 0.166667 0.500000
```

MOTIF PITG_02328_bZIP_1

letter-probability matrix: alength= 4 nsites= 10 E= 0
0.166667 0.333333 0.166667 0.333333
0.250000 0.062500 0.562500 0.125000
0.041667 0.041667 0.041667 0.875000
0.875000 0.041667 0.041667 0.041667
0.875000 0.041667 0.041667 0.041667
0.041667 0.041667 0.041667 0.875000
0.041667 0.041667 0.750000 0.166667
0.875000 0.041667 0.041667 0.041667
0.045455 0.545455 0.045455 0.363636
0.166667 0.083333 0.416667 0.333333

MOTIF PITG_06748_Myb_DNA-binding

letter-probability matrix: alength= 4 nsites= 10 E= 0
0.615385 0.076923 0.230769 0.076923
0.086957 0.782609 0.086957 0.043478
0.250000 0.375000 0.333333 0.041667
0.041667 0.041667 0.875000 0.041667
0.041667 0.041667 0.041667 0.875000
0.041667 0.041667 0.041667 0.875000
0.791667 0.041667 0.041667 0.125000
0.041667 0.875000 0.041667 0.041667
0.600000 0.066667 0.200000 0.133333
0.200000 0.200000 0.200000 0.400000

MOTIF PITG_18417_bZIP_1

letter-probability matrix: alength= 4 nsites= 10 E= 0
0.166667 0.166667 0.166667 0.500000
0.062500 0.187500 0.312500 0.437500
0.636364 0.045455 0.272727 0.045455
0.041667 0.875000 0.041667 0.041667
0.041667 0.041667 0.875000 0.041667
0.041667 0.041667 0.041667 0.875000
0.041667 0.875000 0.041667 0.041667
0.875000 0.041667 0.041667 0.041667
0.045455 0.136364 0.045455 0.772727
0.166667 0.583333 0.083333 0.166667
0.333333 0.166667 0.333333 0.166667

MOTIF PITG_06978_CENP-B_N

```
letter-probability matrix: alength= 4 nsites= 10 E= 0
0.200000 0.200000 0.200000 0.400000
0.111111 0.222222 0.555556 0.111111
0.052632 0.789474 0.052632 0.105263
0.041667 0.041667 0.875000 0.041667
0.333333 0.125000 0.041667 0.500000
0.875000 0.041667 0.041667 0.041667
0.041667 0.875000 0.041667 0.041667
0.041667 0.125000 0.041667 0.791667
0.043478 0.043478 0.043478 0.869565
0.789474 0.052632 0.052632 0.105263
0.444444 0.111111 0.333333 0.111111


MOTIF PITG_13587_bZIP_1

letter-probability matrix: alength= 4 nsites= 10 E= 0
0.166667 0.166667 0.166667 0.500000
0.142857 0.285714 0.428571 0.142857
0.250000 0.125000 0.125000 0.500000
0.500000 0.100000 0.200000 0.200000
0.083333 0.666667 0.083333 0.166667
0.692308 0.076923 0.153846 0.076923
0.230769 0.615385 0.076923 0.076923
0.571429 0.071429 0.214286 0.142857
0.083333 0.083333 0.083333 0.750000
0.090909 0.090909 0.636364 0.181818
0.100000 0.200000 0.100000 0.600000
0.375000 0.125000 0.250000 0.250000
0.333333 0.333333 0.166667 0.166667
0.400000 0.200000 0.200000 0.200000
0.200000 0.200000 0.200000 0.400000


MOTIF PITG_05545_Homeobox

letter-probability matrix: alength= 4 nsites= 10 E= 0
0.400000 0.066667 0.200000 0.333333
0.050000 0.600000 0.200000 0.150000
0.217391 0.173913 0.565217 0.043478
0.041667 0.125000 0.041667 0.791667
0.041667 0.041667 0.875000 0.041667
0.125000 0.041667 0.041667 0.791667
0.041667 0.875000 0.041667 0.041667
0.875000 0.041667 0.041667 0.041667
0.461538 0.230769 0.230769 0.076923
0.125000 0.625000 0.125000 0.125000
0.200000 0.200000 0.400000 0.200000
```

MOTIF PITG_00215_CG-1

letter-probability matrix: alength= 4 nsites= 10 E= 0
0.100000 0.100000 0.300000 0.500000
0.062500 0.062500 0.750000 0.125000
0.041667 0.041667 0.833333 0.083333
0.041667 0.125000 0.791667 0.041667
0.208333 0.083333 0.583333 0.125000
0.041667 0.041667 0.041667 0.875000
0.041667 0.041667 0.750000 0.166667
0.041667 0.041667 0.791667 0.125000
0.388889 0.388889 0.166667 0.055556
0.166667 0.250000 0.333333 0.250000

MOTIF PITG_19429_BrkDBD

letter-probability matrix: alength= 4 nsites= 10 E= 0
0.222222 0.055556 0.111111 0.611111
0.695652 0.043478 0.086957 0.173913
0.541667 0.083333 0.083333 0.291667
0.041667 0.041667 0.083333 0.833333
0.666667 0.041667 0.083333 0.208333
0.458333 0.041667 0.458333 0.041667
0.041667 0.041667 0.041667 0.875000
0.750000 0.041667 0.125000 0.083333
0.100000 0.400000 0.100000 0.400000
0.400000 0.200000 0.200000 0.200000

MOTIF PITG_01135_Homeobox

letter-probability matrix: alength= 4 nsites= 10 E= 0
0.333333 0.166667 0.250000 0.250000
0.052632 0.263158 0.631579 0.052632
0.041667 0.041667 0.041667 0.875000
0.833333 0.041667 0.083333 0.041667
0.041667 0.041667 0.875000 0.041667
0.041667 0.041667 0.041667 0.875000
0.041667 0.875000 0.041667 0.041667
0.875000 0.041667 0.041667 0.041667
0.625000 0.125000 0.062500 0.187500
0.333333 0.222222 0.111111 0.333333

MOTIF PITG_00016_HTH_psq

letter-probability matrix: alength= 4 nsites= 10 E= 0
0.153846 0.230769 0.307692 0.307692
0.041667 0.666667 0.041667 0.250000

0.041667 0.041667 0.875000 0.041667
0.041667 0.041667 0.083333 0.833333
0.791667 0.041667 0.041667 0.125000
0.875000 0.041667 0.041667 0.041667
0.041667 0.875000 0.041667 0.041667
0.875000 0.041667 0.041667 0.041667
0.266667 0.266667 0.333333 0.133333

MOTIF PITG_10557_bZIP_1

letter-probability matrix: alength= 4 nsites= 10 E= 0
0.285714 0.142857 0.428571 0.142857
0.062500 0.062500 0.062500 0.812500
0.041667 0.041667 0.875000 0.041667
0.083333 0.083333 0.041667 0.791667
0.083333 0.041667 0.083333 0.791667
0.041667 0.708333 0.125000 0.125000
0.041667 0.041667 0.833333 0.083333
0.625000 0.041667 0.083333 0.250000
0.333333 0.190476 0.285714 0.190476
0.083333 0.500000 0.166667 0.250000

MOTIF PITG_12841_CENP-B_N

letter-probability matrix: alength= 4 nsites= 10 E= 0
0.200000 0.400000 0.200000 0.200000
0.181818 0.272727 0.363636 0.181818
0.190476 0.095238 0.190476 0.523810
0.043478 0.043478 0.782609 0.130435
0.041667 0.041667 0.041667 0.875000
0.083333 0.041667 0.041667 0.833333
0.833333 0.041667 0.041667 0.083333
0.875000 0.041667 0.041667 0.041667
0.086957 0.826087 0.043478 0.043478
0.588235 0.117647 0.117647 0.176471
0.142857 0.285714 0.285714 0.285714
0.200000 0.200000 0.400000 0.200000

MOTIF PITG_08807_Myb_DNA-binding

letter-probability matrix: alength= 4 nsites= 10 E= 0
0.166667 0.166667 0.500000 0.166667
0.230769 0.076923 0.307692 0.384615
0.043478 0.869565 0.043478 0.043478
0.041667 0.791667 0.125000 0.041667
0.041667 0.041667 0.875000 0.041667
0.041667 0.041667 0.041667 0.875000

0.041667 0.041667 0.041667 0.875000
0.791667 0.041667 0.041667 0.125000
0.045455 0.863636 0.045455 0.045455
0.466667 0.133333 0.200000 0.200000
0.200000 0.200000 0.200000 0.400000

MOTIF PITG_01306_zf-C2H2

letter-probability matrix: alength= 4 nsites= 10 E= 0
0.200000 0.200000 0.400000 0.200000
0.142857 0.142857 0.571429 0.142857
0.333333 0.333333 0.111111 0.222222
0.235294 0.235294 0.176471 0.352941
0.090909 0.045455 0.818182 0.045455
0.086957 0.043478 0.043478 0.826087
0.083333 0.041667 0.833333 0.041667
0.041667 0.833333 0.083333 0.041667
0.826087 0.043478 0.086957 0.043478
0.047619 0.714286 0.095238 0.142857
0.473684 0.052632 0.368421 0.105263
0.363636 0.272727 0.181818 0.181818
0.333333 0.166667 0.166667 0.333333
0.200000 0.200000 0.400000 0.200000

MOTIF PITG_08755_Myb_DNA-binding

letter-probability matrix: alength= 4 nsites= 10 E= 0
0.363636 0.090909 0.363636 0.181818
0.041667 0.875000 0.041667 0.041667
0.166667 0.625000 0.166667 0.041667
0.041667 0.041667 0.875000 0.041667
0.041667 0.041667 0.041667 0.875000
0.041667 0.041667 0.041667 0.875000
0.750000 0.041667 0.041667 0.166667
0.041667 0.875000 0.041667 0.041667
0.411765 0.117647 0.352941 0.117647

MOTIF PITG_09816_bZIP_1

letter-probability matrix: alength= 4 nsites= 10 E= 0
0.285714 0.428571 0.142857 0.142857
0.500000 0.071429 0.214286 0.214286
0.761905 0.142857 0.047619 0.047619
0.833333 0.041667 0.041667 0.083333
0.083333 0.041667 0.041667 0.833333
0.875000 0.041667 0.041667 0.041667
0.041667 0.041667 0.041667 0.875000

```
0.666667 0.041667 0.041667 0.250000
0.095238 0.238095 0.047619 0.619048
0.285714 0.214286 0.285714 0.214286
0.285714 0.142857 0.142857 0.428571


MOTIF PITG_19851_Myb_DNA-binding

letter-probability matrix: alength= 4 nsites= 10 E= 0
0.333333 0.083333 0.166667 0.416667
0.736842 0.052632 0.105263 0.105263
0.041667 0.875000 0.041667 0.041667
0.041667 0.041667 0.041667 0.875000
0.041667 0.041667 0.875000 0.041667
0.750000 0.041667 0.125000 0.083333
0.833333 0.083333 0.041667 0.041667
0.041667 0.875000 0.041667 0.041667
0.312500 0.125000 0.125000 0.437500
0.111111 0.444444 0.111111 0.333333


MOTIF PITG_17750_zf-C2H2

letter-probability matrix: alength= 4 nsites= 10 E= 0
0.200000 0.100000 0.100000 0.600000
0.055556 0.055556 0.833333 0.055556
0.041667 0.875000 0.041667 0.041667
0.750000 0.166667 0.041667 0.041667
0.500000 0.333333 0.125000 0.041667
0.875000 0.041667 0.041667 0.041667
0.041667 0.041667 0.041667 0.875000
0.041667 0.875000 0.041667 0.041667
0.222222 0.388889 0.111111 0.277778
0.100000 0.200000 0.100000 0.600000


MOTIF PITG_18326_HSF_DNA-bind

letter-probability matrix: alength= 4 nsites= 10 E= 0
0.285714 0.142857 0.285714 0.285714
0.333333 0.083333 0.083333 0.500000
0.166667 0.111111 0.055556 0.666667
0.090909 0.090909 0.045455 0.772727
0.045455 0.045455 0.045455 0.863636
0.041667 0.041667 0.041667 0.875000
0.041667 0.833333 0.041667 0.083333
0.041667 0.708333 0.041667 0.208333
0.857143 0.047619 0.047619 0.047619
0.062500 0.437500 0.062500 0.437500
0.300000 0.100000 0.500000 0.100000
```

0.166667 0.166667 0.500000 0.166667
0.500000 0.166667 0.166667 0.166667

MOTIF PITG_15175_CENP-B_N

letter-probability matrix: alength= 4 nsites= 10 E= 0
0.300000 0.300000 0.300000 0.100000
0.043478 0.043478 0.826087 0.086957
0.166667 0.208333 0.458333 0.166667
0.041667 0.041667 0.041667 0.875000
0.041667 0.041667 0.041667 0.875000
0.708333 0.041667 0.041667 0.208333
0.875000 0.041667 0.041667 0.041667
0.041667 0.875000 0.041667 0.041667
0.777778 0.055556 0.111111 0.055556
0.400000 0.200000 0.200000 0.200000

MOTIF PITG_11760_HSF_DNA-bind

letter-probability matrix: alength= 4 nsites= 10 E= 0
0.461538 0.153846 0.153846 0.230769
0.333333 0.095238 0.095238 0.476190
0.130435 0.043478 0.043478 0.782609
0.041667 0.125000 0.041667 0.791667
0.083333 0.750000 0.125000 0.041667
0.125000 0.041667 0.791667 0.041667
0.791667 0.041667 0.041667 0.125000
0.875000 0.041667 0.041667 0.041667
0.466667 0.133333 0.066667 0.333333
0.428571 0.142857 0.142857 0.285714
0.200000 0.200000 0.200000 0.400000

MOTIF PITG_15721_Homeobox

letter-probability matrix: alength= 4 nsites= 10 E= 0
0.200000 0.200000 0.200000 0.400000
0.095238 0.047619 0.809524 0.047619
0.083333 0.666667 0.125000 0.125000
0.125000 0.333333 0.208333 0.333333
0.541667 0.083333 0.041667 0.333333
0.625000 0.041667 0.291667 0.041667
0.041667 0.041667 0.083333 0.833333
0.041667 0.875000 0.041667 0.041667
0.869565 0.043478 0.043478 0.043478
0.428571 0.142857 0.142857 0.285714

MOTIF PITG_17861_BrkDBD

letter-probability matrix: alength= 4 nsites= 10 E= 0
0.571429 0.142857 0.142857 0.142857
0.266667 0.266667 0.133333 0.333333
0.043478 0.826087 0.086957 0.043478
0.041667 0.875000 0.041667 0.041667
0.041667 0.041667 0.875000 0.041667
0.041667 0.875000 0.041667 0.041667
0.041667 0.875000 0.041667 0.041667
0.875000 0.041667 0.041667 0.041667
0.047619 0.333333 0.047619 0.571429
0.230769 0.384615 0.230769 0.153846
0.200000 0.400000 0.200000 0.200000

MOTIF PITG_01388_zf-C2H2

letter-probability matrix: alength= 4 nsites= 10 E= 0
0.571429 0.142857 0.142857 0.142857
0.642857 0.071429 0.142857 0.142857
0.650000 0.100000 0.050000 0.200000
0.695652 0.043478 0.173913 0.086957
0.043478 0.043478 0.043478 0.869565
0.041667 0.041667 0.875000 0.041667
0.666667 0.208333 0.041667 0.083333
0.208333 0.041667 0.041667 0.708333
0.190476 0.523810 0.047619 0.238095
0.500000 0.285714 0.142857 0.071429
0.375000 0.125000 0.125000 0.375000
0.400000 0.200000 0.200000 0.200000
0.200000 0.400000 0.200000 0.200000

MOTIF PITG_04694_HSF_DNA-bind

letter-probability matrix: alength= 4 nsites= 10 E= 0
0.285714 0.142857 0.142857 0.428571
0.285714 0.142857 0.071429 0.500000
0.105263 0.105263 0.052632 0.736842
0.571429 0.142857 0.095238 0.190476
0.041667 0.041667 0.041667 0.875000
0.333333 0.166667 0.458333 0.041667
0.041667 0.041667 0.875000 0.041667
0.875000 0.041667 0.041667 0.041667
0.857143 0.047619 0.047619 0.047619
0.714286 0.071429 0.142857 0.071429
0.333333 0.111111 0.444444 0.111111
0.142857 0.142857 0.285714 0.428571

MOTIF PITG_16038_bZIP_1

letter-probability matrix: alength= 4 nsites= 10 E= 0
0.200000 0.200000 0.200000 0.400000
0.166667 0.083333 0.083333 0.666667
0.400000 0.266667 0.200000 0.133333
0.095238 0.142857 0.047619 0.714286
0.041667 0.041667 0.875000 0.041667
0.041667 0.041667 0.041667 0.875000
0.875000 0.041667 0.041667 0.041667
0.875000 0.041667 0.041667 0.041667
0.043478 0.043478 0.043478 0.869565
0.312500 0.187500 0.187500 0.312500
0.461538 0.153846 0.153846 0.230769
0.142857 0.428571 0.142857 0.285714

MOTIF PITG_08199_HSF_DNA-bind

letter-probability matrix: alength= 4 nsites= 10 E= 0
0.272727 0.181818 0.272727 0.272727
0.631579 0.157895 0.157895 0.052632
0.083333 0.041667 0.833333 0.041667
0.833333 0.083333 0.041667 0.041667
0.875000 0.041667 0.041667 0.041667
0.041667 0.041667 0.041667 0.875000
0.041667 0.041667 0.041667 0.875000
0.041667 0.875000 0.041667 0.041667
0.058824 0.529412 0.235294 0.176471
0.222222 0.222222 0.222222 0.333333

MOTIF PITG_12760_HLH

letter-probability matrix: alength= 4 nsites= 10 E= 0
0.636364 0.090909 0.181818 0.090909
0.700000 0.200000 0.050000 0.050000
0.041667 0.875000 0.041667 0.041667
0.875000 0.041667 0.041667 0.041667
0.041667 0.041667 0.375000 0.541667
0.708333 0.208333 0.041667 0.041667
0.041667 0.041667 0.041667 0.875000
0.041667 0.041667 0.875000 0.041667
0.058824 0.058824 0.705882 0.176471
0.125000 0.250000 0.125000 0.500000

MOTIF PITG_14515_zf-C2H2

```
letter-probability matrix: alength= 4 nsites= 10 E= 0
0.333333 0.333333 0.111111 0.222222
0.133333 0.066667 0.733333 0.066667
0.043478 0.869565 0.043478 0.043478
0.041667 0.833333 0.041667 0.083333
0.041667 0.875000 0.041667 0.041667
0.875000 0.041667 0.041667 0.041667
0.041667 0.041667 0.041667 0.875000
0.041667 0.875000 0.041667 0.041667
0.210526 0.473684 0.052632 0.263158
0.076923 0.615385 0.076923 0.230769
0.200000 0.400000 0.200000 0.200000


MOTIF PITG_00015_HTH_psq

letter-probability matrix: alength= 4 nsites= 10 E= 0
0.153846 0.230769 0.307692 0.307692
0.041667 0.625000 0.041667 0.291667
0.041667 0.041667 0.875000 0.041667
0.041667 0.041667 0.041667 0.875000
0.833333 0.041667 0.041667 0.083333
0.875000 0.041667 0.041667 0.041667
0.041667 0.875000 0.041667 0.041667
0.875000 0.041667 0.041667 0.041667
0.266667 0.266667 0.266667 0.200000


MOTIF PITG_09280_bZIP_1

letter-probability matrix: alength= 4 nsites= 10 E= 0
0.200000 0.200000 0.400000 0.200000
0.400000 0.200000 0.200000 0.200000
0.384615 0.230769 0.076923 0.307692
0.052632 0.578947 0.210526 0.157895
0.875000 0.041667 0.041667 0.041667
0.041667 0.875000 0.041667 0.041667
0.041667 0.041667 0.875000 0.041667
0.041667 0.041667 0.041667 0.875000
0.043478 0.043478 0.869565 0.043478
0.043478 0.043478 0.608696 0.304348
0.066667 0.800000 0.066667 0.066667
0.444444 0.222222 0.222222 0.111111


MOTIF PITG_08159_bZIP_1

letter-probability matrix: alength= 4 nsites= 10 E= 0
0.111111 0.333333 0.444444 0.111111
0.176471 0.588235 0.058824 0.176471
```

0.041667 0.041667 0.875000 0.041667
0.083333 0.041667 0.041667 0.833333
0.041667 0.041667 0.083333 0.833333
0.875000 0.041667 0.041667 0.041667
0.083333 0.041667 0.041667 0.833333
0.041667 0.875000 0.041667 0.041667
0.736842 0.105263 0.105263 0.052632
0.272727 0.363636 0.181818 0.181818

MOTIF PITG_10442_CSD

letter-probability matrix: alength= 4 nsites= 10 E= 0
0.250000 0.250000 0.250000 0.250000
0.045455 0.045455 0.772727 0.136364
0.041667 0.875000 0.041667 0.041667
0.875000 0.041667 0.041667 0.041667
0.083333 0.041667 0.833333 0.041667
0.041667 0.833333 0.041667 0.083333
0.041667 0.041667 0.041667 0.875000
0.041667 0.041667 0.875000 0.041667
0.437500 0.250000 0.125000 0.187500
0.166667 0.500000 0.166667 0.166667

MOTIF PITG_00988_Myb_DNA-binding

letter-probability matrix: alength= 4 nsites= 10 E= 0
0.200000 0.200000 0.400000 0.200000
0.200000 0.300000 0.100000 0.400000
0.750000 0.050000 0.150000 0.050000
0.652174 0.043478 0.260870 0.043478
0.041667 0.875000 0.041667 0.041667
0.166667 0.750000 0.041667 0.041667
0.041667 0.041667 0.875000 0.041667
0.041667 0.041667 0.083333 0.833333
0.043478 0.043478 0.043478 0.869565
0.666667 0.055556 0.055556 0.222222
0.125000 0.500000 0.125000 0.250000
0.400000 0.200000 0.200000 0.200000

MOTIF PITG_16183_bZIP_1

letter-probability matrix: alength= 4 nsites= 10 E= 0
0.200000 0.200000 0.200000 0.400000
0.142857 0.285714 0.428571 0.142857
0.500000 0.166667 0.083333 0.250000
0.071429 0.071429 0.571429 0.285714
0.071429 0.142857 0.071429 0.714286

```
0.285714 0.071429 0.571429 0.071429
0.571429 0.214286 0.071429 0.142857
0.214286 0.071429 0.142857 0.571429
0.076923 0.692308 0.153846 0.076923
0.636364 0.090909 0.181818 0.090909
0.333333 0.333333 0.166667 0.166667


MOTIF PITG_22459_HSF_DNA-bind

letter-probability matrix: alength= 4 nsites= 10 E= 0
0.500000 0.062500 0.062500 0.375000
0.190476 0.047619 0.095238 0.666667
0.173913 0.043478 0.043478 0.739130
0.090909 0.045455 0.045455 0.818182
0.217391 0.652174 0.086957 0.043478
0.043478 0.043478 0.869565 0.043478
0.869565 0.043478 0.043478 0.043478
0.833333 0.041667 0.041667 0.083333
0.230769 0.230769 0.076923 0.461538
0.250000 0.125000 0.125000 0.500000
0.166667 0.500000 0.166667 0.166667
0.166667 0.333333 0.166667 0.333333


MOTIF PITG_01305_zf-C2H2

letter-probability matrix: alength= 4 nsites= 10 E= 0
0.142857 0.142857 0.428571 0.285714
0.230769 0.153846 0.461538 0.153846
0.350000 0.300000 0.150000 0.200000
0.166667 0.250000 0.125000 0.458333
0.208333 0.041667 0.666667 0.083333
0.041667 0.041667 0.041667 0.875000
0.041667 0.041667 0.875000 0.041667
0.041667 0.833333 0.041667 0.083333
0.809524 0.047619 0.047619 0.095238
0.066667 0.666667 0.066667 0.200000
0.375000 0.250000 0.250000 0.125000


MOTIF PITG_10768_zf-C2H2

letter-probability matrix: alength= 4 nsites= 10 E= 0
0.625000 0.125000 0.125000 0.125000
0.266667 0.133333 0.400000 0.200000
0.041667 0.041667 0.875000 0.041667
0.875000 0.041667 0.041667 0.041667
0.041667 0.041667 0.041667 0.875000
0.041667 0.041667 0.041667 0.875000
```

```
0.041667 0.041667 0.041667 0.875000
0.041667 0.041667 0.875000 0.041667
0.150000 0.700000 0.050000 0.100000
0.384615 0.230769 0.230769 0.153846


MOTIF PITG_01080_Homeobox

letter-probability matrix: alength= 4 nsites= 10 E= 0
0.500000 0.100000 0.300000 0.100000
0.058824 0.588235 0.294118 0.058824
0.045455 0.863636 0.045455 0.045455
0.041667 0.875000 0.041667 0.041667
0.875000 0.041667 0.041667 0.041667
0.041667 0.041667 0.041667 0.875000
0.041667 0.875000 0.041667 0.041667
0.875000 0.041667 0.041667 0.041667
0.611111 0.166667 0.055556 0.166667
0.363636 0.090909 0.090909 0.454545
0.166667 0.500000 0.166667 0.166667


MOTIF PITG_07059_MADs

letter-probability matrix: alength= 4 nsites= 10 E= 0
0.050000 0.200000 0.050000 0.700000
0.833333 0.041667 0.041667 0.083333
0.500000 0.041667 0.041667 0.416667
0.708333 0.041667 0.041667 0.208333
0.625000 0.041667 0.041667 0.291667
0.833333 0.041667 0.041667 0.083333
0.041667 0.041667 0.041667 0.875000
0.875000 0.041667 0.041667 0.041667
0.125000 0.125000 0.500000 0.250000


MOTIF PITG_17673_HLH

letter-probability matrix: alength= 4 nsites= 10 E= 0
0.500000 0.166667 0.166667 0.166667
0.250000 0.083333 0.583333 0.083333
0.263158 0.578947 0.052632 0.105263
0.875000 0.041667 0.041667 0.041667
0.041667 0.833333 0.041667 0.083333
0.125000 0.041667 0.791667 0.041667
0.041667 0.083333 0.041667 0.833333
0.083333 0.083333 0.666667 0.166667
0.090909 0.727273 0.045455 0.136364
0.187500 0.500000 0.062500 0.250000
0.333333 0.222222 0.111111 0.333333
```

```
MOTIF PITG_10525_zf-C2H2

letter-probability matrix: alength= 4 nsites= 10 E= 0
0.142857 0.571429 0.142857 0.142857
0.125000 0.125000 0.625000 0.125000
0.176471 0.529412 0.058824 0.235294
0.041667 0.875000 0.041667 0.041667
0.041667 0.041667 0.625000 0.291667
0.041667 0.125000 0.041667 0.791667
0.041667 0.875000 0.041667 0.041667
0.041667 0.041667 0.875000 0.041667
0.047619 0.047619 0.857143 0.047619
0.850000 0.050000 0.050000 0.050000
0.272727 0.090909 0.181818 0.454545


MOTIF PITG_05353_HSF_DNA-bind

letter-probability matrix: alength= 4 nsites= 10 E= 0
0.400000 0.200000 0.200000 0.200000
0.500000 0.166667 0.166667 0.166667
0.538462 0.076923 0.153846 0.230769
0.300000 0.050000 0.100000 0.550000
0.086957 0.043478 0.043478 0.826087
0.041667 0.083333 0.041667 0.833333
0.125000 0.750000 0.041667 0.083333
0.083333 0.041667 0.833333 0.041667
0.869565 0.043478 0.043478 0.043478
0.863636 0.045455 0.045455 0.045455
0.600000 0.133333 0.066667 0.200000
0.500000 0.125000 0.125000 0.250000
0.400000 0.200000 0.200000 0.200000


MOTIF PITG_12296_HTH_psq

letter-probability matrix: alength= 4 nsites= 10 E= 0
0.142857 0.214286 0.357143 0.285714
0.043478 0.652174 0.043478 0.260870
0.041667 0.041667 0.875000 0.041667
0.041667 0.041667 0.041667 0.875000
0.875000 0.041667 0.041667 0.041667
0.875000 0.041667 0.041667 0.041667
0.041667 0.875000 0.041667 0.041667
0.875000 0.041667 0.041667 0.041667
0.285714 0.214286 0.357143 0.142857
0.200000 0.200000 0.200000 0.400000
```

MOTIF PITG_05990_Myb_DNA-binding

letter-probability matrix: alength= 4 nsites= 10 E= 0
0.333333 0.111111 0.444444 0.111111
0.823529 0.058824 0.058824 0.058824
0.571429 0.047619 0.333333 0.047619
0.583333 0.041667 0.041667 0.333333
0.875000 0.041667 0.041667 0.041667
0.083333 0.041667 0.041667 0.833333
0.041667 0.833333 0.041667 0.083333
0.041667 0.875000 0.041667 0.041667
0.842105 0.052632 0.052632 0.052632
0.090909 0.090909 0.727273 0.090909
0.142857 0.142857 0.142857 0.571429

MOTIF PITG_01387_zf-C2H2

letter-probability matrix: alength= 4 nsites= 10 E= 0
0.444444 0.333333 0.111111 0.111111
0.235294 0.176471 0.352941 0.235294
0.095238 0.047619 0.761905 0.095238
0.250000 0.041667 0.666667 0.041667
0.875000 0.041667 0.041667 0.041667
0.083333 0.041667 0.041667 0.833333
0.083333 0.625000 0.125000 0.166667
0.083333 0.791667 0.041667 0.083333
0.263158 0.157895 0.263158 0.315789
0.363636 0.090909 0.181818 0.363636
0.285714 0.285714 0.142857 0.285714

MOTIF PITG_04902_zf-C2H2

letter-probability matrix: alength= 4 nsites= 10 E= 0
0.333333 0.166667 0.166667 0.333333
0.411765 0.058824 0.058824 0.470588
0.708333 0.041667 0.083333 0.166667
0.208333 0.083333 0.041667 0.666667
0.625000 0.041667 0.125000 0.208333
0.458333 0.083333 0.041667 0.416667
0.875000 0.041667 0.041667 0.041667
0.125000 0.166667 0.041667 0.666667
0.500000 0.045455 0.045455 0.409091
0.545455 0.090909 0.090909 0.272727

MOTIF PITG_05989_Myb_DNA-binding

letter-probability matrix: alength= 4 nsites= 10 E= 0
0.700000 0.100000 0.100000 0.100000
0.052632 0.789474 0.052632 0.105263
0.041667 0.041667 0.041667 0.875000
0.041667 0.041667 0.583333 0.333333
0.125000 0.041667 0.791667 0.041667
0.875000 0.041667 0.041667 0.041667
0.083333 0.041667 0.041667 0.833333
0.166667 0.041667 0.041667 0.750000
0.055556 0.555556 0.055556 0.333333
0.555556 0.111111 0.111111 0.222222

MOTIF PITG_19177_zf-C2H2

letter-probability matrix: alength= 4 nsites= 10 E= 0
0.357143 0.214286 0.357143 0.071429
0.523810 0.047619 0.095238 0.333333
0.041667 0.041667 0.875000 0.041667
0.875000 0.041667 0.041667 0.041667
0.041667 0.041667 0.875000 0.041667
0.041667 0.041667 0.041667 0.875000
0.041667 0.041667 0.875000 0.041667
0.875000 0.041667 0.041667 0.041667
0.214286 0.142857 0.071429 0.571429
0.142857 0.142857 0.428571 0.285714

MOTIF PITG_03306_HSF_DNA-bind

letter-probability matrix: alength= 4 nsites= 10 E= 0
0.125000 0.125000 0.500000 0.250000
0.312500 0.062500 0.562500 0.062500
0.125000 0.041667 0.041667 0.791667
0.125000 0.041667 0.041667 0.791667
0.125000 0.791667 0.041667 0.041667
0.125000 0.541667 0.041667 0.291667
0.041667 0.041667 0.625000 0.291667
0.541667 0.041667 0.083333 0.333333
0.650000 0.050000 0.050000 0.250000
0.333333 0.083333 0.083333 0.500000

MOTIF PITG_06416_E2F_TDP

letter-probability matrix: alength= 4 nsites= 10 E= 0
0.166667 0.166667 0.333333 0.333333
0.125000 0.250000 0.125000 0.500000
0.083333 0.083333 0.166667 0.666667
0.045455 0.681818 0.227273 0.045455

0.041667 0.375000 0.541667 0.041667
0.041667 0.875000 0.041667 0.041667
0.041667 0.041667 0.875000 0.041667
0.041667 0.750000 0.166667 0.041667
0.045455 0.545455 0.363636 0.045455
0.800000 0.100000 0.050000 0.050000
0.562500 0.125000 0.125000 0.187500
0.500000 0.166667 0.166667 0.166667

MOTIF PITG_17894_zf-C2H2

letter-probability matrix: alength= 4 nsites= 10 E= 0
0.400000 0.200000 0.200000 0.200000
0.200000 0.200000 0.200000 0.400000
0.090909 0.272727 0.272727 0.363636
0.636364 0.136364 0.136364 0.090909
0.125000 0.041667 0.666667 0.166667
0.041667 0.083333 0.708333 0.166667
0.750000 0.041667 0.166667 0.041667
0.041667 0.166667 0.041667 0.750000
0.652174 0.217391 0.086957 0.043478
0.608696 0.217391 0.086957 0.086957
0.117647 0.176471 0.294118 0.411765
0.500000 0.166667 0.166667 0.166667

MOTIF PITG_15534_CENP-B_N

letter-probability matrix: alength= 4 nsites= 10 E= 0
0.125000 0.250000 0.250000 0.375000
0.176471 0.058824 0.588235 0.176471
0.041667 0.041667 0.041667 0.875000
0.041667 0.041667 0.041667 0.875000
0.041667 0.041667 0.041667 0.875000
0.875000 0.041667 0.041667 0.041667
0.875000 0.041667 0.041667 0.041667
0.041667 0.875000 0.041667 0.041667
0.700000 0.050000 0.150000 0.100000
0.272727 0.272727 0.181818 0.272727

MOTIF PITG_10769_zf-C2H2

letter-probability matrix: alength= 4 nsites= 10 E= 0
0.125000 0.125000 0.125000 0.625000
0.066667 0.066667 0.666667 0.200000
0.041667 0.875000 0.041667 0.041667
0.541667 0.375000 0.041667 0.041667
0.333333 0.541667 0.083333 0.041667

0.875000 0.041667 0.041667 0.041667
0.041667 0.041667 0.041667 0.875000
0.041667 0.875000 0.041667 0.041667
0.050000 0.700000 0.050000 0.200000
0.076923 0.307692 0.076923 0.538462

MOTIF PITG_08960_Myb_DNA-binding

letter-probability matrix: alength= 4 nsites= 10 E= 0
0.181818 0.272727 0.272727 0.272727
0.833333 0.055556 0.055556 0.055556
0.043478 0.869565 0.043478 0.043478
0.041667 0.875000 0.041667 0.041667
0.041667 0.041667 0.875000 0.041667
0.041667 0.041667 0.875000 0.041667
0.041667 0.166667 0.041667 0.750000
0.041667 0.875000 0.041667 0.041667
0.411765 0.058824 0.470588 0.058824
0.100000 0.400000 0.100000 0.400000
0.200000 0.200000 0.400000 0.200000

MOTIF PITG_12584_HLH

letter-probability matrix: alength= 4 nsites= 10 E= 0
0.600000 0.100000 0.200000 0.100000
0.684211 0.210526 0.052632 0.052632
0.041667 0.875000 0.041667 0.041667
0.875000 0.041667 0.041667 0.041667
0.041667 0.041667 0.291667 0.625000
0.791667 0.125000 0.041667 0.041667
0.041667 0.041667 0.041667 0.875000
0.041667 0.041667 0.875000 0.041667
0.055556 0.055556 0.722222 0.166667
0.333333 0.222222 0.111111 0.333333

MOTIF PITG_07057_HLH

letter-probability matrix: alength= 4 nsites= 10 E= 0
0.166667 0.166667 0.500000 0.166667
0.588235 0.058824 0.294118 0.058824
0.041667 0.875000 0.041667 0.041667
0.750000 0.041667 0.166667 0.041667
0.041667 0.875000 0.041667 0.041667
0.041667 0.041667 0.875000 0.041667
0.041667 0.375000 0.041667 0.541667
0.041667 0.041667 0.875000 0.041667
0.045455 0.590909 0.045455 0.318182

```
0.090909 0.636364 0.181818 0.090909


MOTIF PITG_04701_HSF_DNA-bind

letter-probability matrix: alength= 4 nsites= 10 E= 0
0.200000 0.200000 0.400000 0.200000
0.500000 0.166667 0.166667 0.166667
0.428571 0.142857 0.285714 0.142857
0.125000 0.375000 0.125000 0.375000
0.466667 0.133333 0.333333 0.066667
0.130435 0.043478 0.043478 0.782609
0.041667 0.041667 0.041667 0.875000
0.041667 0.833333 0.041667 0.083333
0.043478 0.173913 0.260870 0.521739
0.818182 0.045455 0.090909 0.045455
0.190476 0.047619 0.714286 0.047619
0.800000 0.050000 0.050000 0.100000
0.769231 0.076923 0.076923 0.076923
0.400000 0.200000 0.200000 0.200000


MOTIF PITG_13133_Myb_DNA-binding

letter-probability matrix: alength= 4 nsites= 10 E= 0
0.750000 0.083333 0.083333 0.083333
0.058824 0.058824 0.470588 0.411765
0.875000 0.041667 0.041667 0.041667
0.041667 0.041667 0.250000 0.666667
0.500000 0.416667 0.041667 0.041667
0.041667 0.875000 0.041667 0.041667
0.166667 0.041667 0.750000 0.041667
0.041667 0.041667 0.875000 0.041667
0.562500 0.062500 0.062500 0.312500
0.090909 0.181818 0.272727 0.454545


MOTIF PITG_10815_zf-C2H2

letter-probability matrix: alength= 4 nsites= 10 E= 0
0.222222 0.222222 0.111111 0.444444
0.142857 0.071429 0.714286 0.071429
0.043478 0.869565 0.043478 0.043478
0.041667 0.750000 0.125000 0.083333
0.041667 0.875000 0.041667 0.041667
0.875000 0.041667 0.041667 0.041667
0.041667 0.041667 0.041667 0.875000
0.041667 0.875000 0.041667 0.041667
0.210526 0.473684 0.052632 0.263158
0.071429 0.714286 0.071429 0.142857
```

```
0.200000 0.400000 0.200000 0.200000


MOTIF PITG_19220_Homeobox

letter-probability matrix: alength= 4 nsites= 10 E= 0
0.125000 0.625000 0.125000 0.125000
0.538462 0.307692 0.076923 0.076923
0.400000 0.050000 0.100000 0.450000
0.333333 0.041667 0.041667 0.583333
0.625000 0.041667 0.041667 0.291667
0.833333 0.041667 0.083333 0.041667
0.041667 0.041667 0.041667 0.875000
0.125000 0.041667 0.791667 0.041667
0.150000 0.250000 0.100000 0.500000
0.733333 0.066667 0.066667 0.133333
0.125000 0.250000 0.125000 0.500000


MOTIF PITG_01056_Myb_DNA-binding

letter-probability matrix: alength= 4 nsites= 10 E= 0
0.285714 0.142857 0.428571 0.142857
0.333333 0.066667 0.400000 0.200000
0.043478 0.869565 0.043478 0.043478
0.041667 0.458333 0.458333 0.041667
0.041667 0.041667 0.875000 0.041667
0.041667 0.041667 0.041667 0.875000
0.041667 0.041667 0.041667 0.875000
0.833333 0.041667 0.041667 0.083333
0.047619 0.857143 0.047619 0.047619
0.538462 0.076923 0.230769 0.153846
0.200000 0.200000 0.200000 0.400000


MOTIF PITG_02480_zf-C2H2

letter-probability matrix: alength= 4 nsites= 10 E= 0
0.200000 0.200000 0.200000 0.400000
0.100000 0.200000 0.100000 0.600000
0.052632 0.052632 0.315789 0.578947
0.041667 0.875000 0.041667 0.041667
0.875000 0.041667 0.041667 0.041667
0.041667 0.875000 0.041667 0.041667
0.041667 0.041667 0.875000 0.041667
0.041667 0.041667 0.041667 0.875000
0.043478 0.739130 0.173913 0.043478
0.611111 0.166667 0.166667 0.055556
0.444444 0.222222 0.111111 0.222222
```

```
MOTIF PITG_11783_HLH

letter-probability matrix: alength= 4 nsites= 10 E= 0
0.500000 0.166667 0.166667 0.166667
0.214286 0.142857 0.428571 0.214286
0.041667 0.875000 0.041667 0.041667
0.875000 0.041667 0.041667 0.041667
0.083333 0.041667 0.833333 0.041667
0.041667 0.875000 0.041667 0.041667
0.041667 0.041667 0.041667 0.875000
0.041667 0.041667 0.875000 0.041667
0.227273 0.636364 0.045455 0.090909
0.214286 0.428571 0.214286 0.142857


MOTIF PITG_20387_HSF_DNA-bind

letter-probability matrix: alength= 4 nsites= 10 E= 0
0.333333 0.166667 0.333333 0.166667
0.500000 0.166667 0.166667 0.166667
0.500000 0.166667 0.166667 0.166667
0.461538 0.076923 0.076923 0.384615
0.125000 0.187500 0.062500 0.625000
0.050000 0.250000 0.100000 0.600000
0.041667 0.041667 0.041667 0.875000
0.041667 0.083333 0.041667 0.833333
0.045455 0.863636 0.045455 0.045455
0.045455 0.636364 0.136364 0.181818
0.863636 0.045455 0.045455 0.045455
0.133333 0.200000 0.066667 0.600000
0.666667 0.083333 0.083333 0.166667
0.375000 0.125000 0.125000 0.375000


MOTIF PITG_02733_bZIP_1

letter-probability matrix: alength= 4 nsites= 10 E= 0
0.142857 0.285714 0.142857 0.428571
0.058824 0.352941 0.411765 0.176471
0.875000 0.041667 0.041667 0.041667
0.041667 0.875000 0.041667 0.041667
0.041667 0.041667 0.875000 0.041667
0.041667 0.041667 0.041667 0.875000
0.041667 0.875000 0.041667 0.041667
0.875000 0.041667 0.041667 0.041667
0.047619 0.571429 0.047619 0.333333
0.090909 0.363636 0.272727 0.272727
```

MOTIF PITG_23160_AP2

letter-probability matrix: alength= 4 nsites= 10 E= 0
0.500000 0.125000 0.125000 0.250000
0.500000 0.041667 0.041667 0.416667
0.583333 0.083333 0.041667 0.291667
0.458333 0.125000 0.041667 0.375000
0.791667 0.041667 0.083333 0.083333
0.333333 0.041667 0.041667 0.583333
0.750000 0.041667 0.125000 0.083333
0.375000 0.041667 0.041667 0.541667
0.450000 0.100000 0.050000 0.400000

Fig. S5. Uncropped gel images

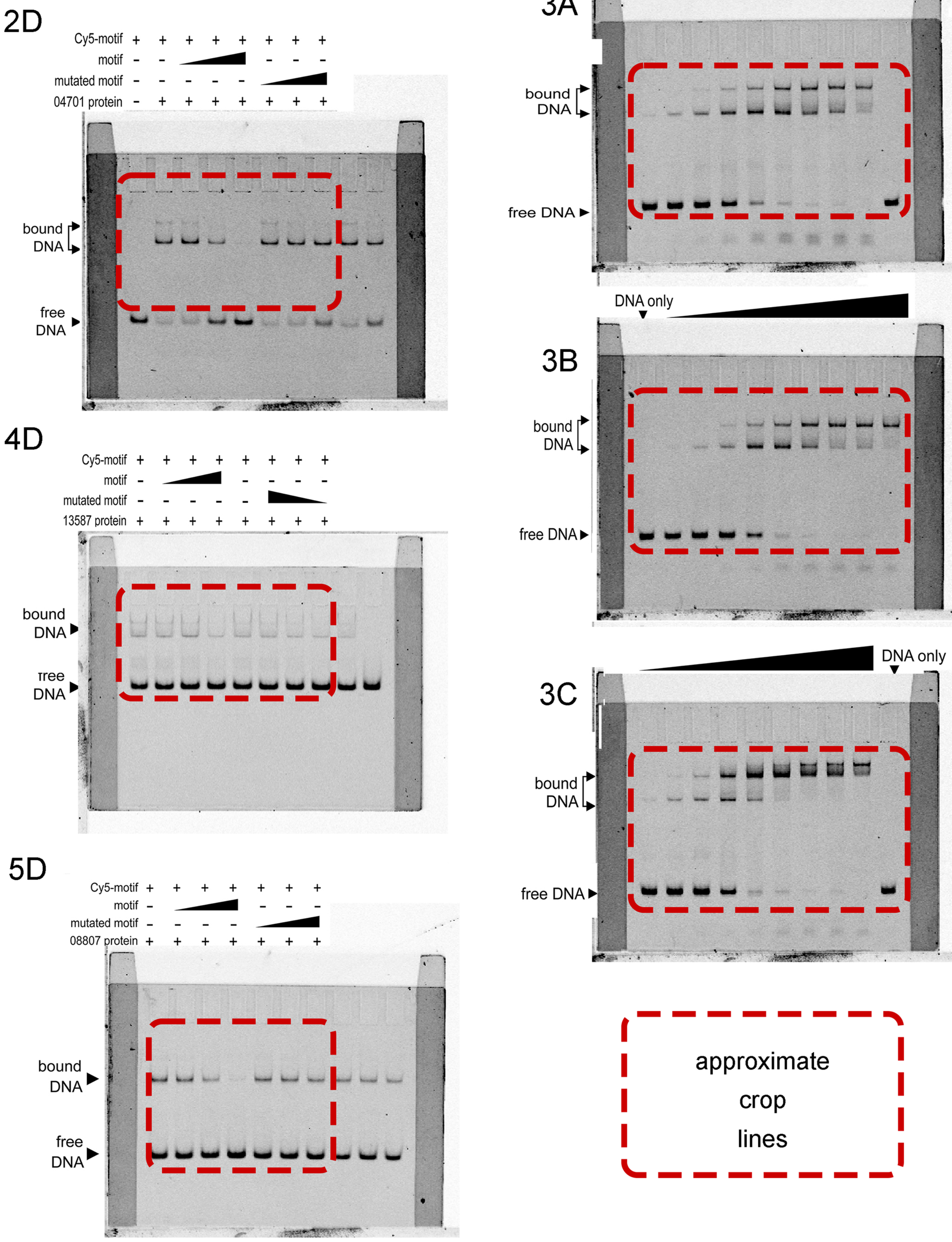

TABLE S2. DNA sequences and DNA/protein concentrations used in EMSA.

Application	Protein	Oligo sequence (only upper strand is shown; motif and mutated motifs are is in bold)	Protein concentration(s)	Oligo concentration	Notes
EMSA competitor assay	Pi13587-bZIP	5'-ATGCCCGTCTCTAACACGTGTCCTCTCGATACTA	40 nM	30 nM	wild-type motif
EMSA competitor assay	Pi13587-bZIP	5'-ATGCCCGTCTCTACTAGTCCTCCTCTCGATACTA	"	"	mutated version of above
EMSA competitor assay	Pi08807-Myb	5'-CTACACTATCATTCTCCGTTACGTCCTACTGTATTGG	200 nM	"	wild-type motif
EMSA competitor assay	Pi08807-Myb	5'-CTACACTATCATTAGACTGTCATCCCTACTGTATTGG	"	"	mutated version of above
EMSA competitor assay	Pi04701-HSF	5'-TAAATATAGCACGCTTCTAGAAACTGCAGCACAACA	40 nM	"	wild-type motif
EMSA competitor assay	Pi04701-HSF	5-TAAATATAGACATGGGAATTCCCAGTAAGCACAACA	"	"	mutated version of above
EMSA titration assay	Pi04701-HSF	5'-TATACCAGCTGCTCATTTTCCATTTGGCCGCATCCC	0,3,10,30,100,200,400 ,800,1000,2000 nM	20 nM	for testing binding to one site of nTTCn
EMSA titration assay	Pi04701-HSF	5'-TAAATATAGCACGCTTCTAGAAACTGCAGCACAACA	"	"	for testing binding to two sites of nTTCn
EMSA titration assay	Pi04701-HSF	5'-TAAATATAGCACGCGAATATTCTAGAAACTGCAGCACAACA	"	"	for testing binding to three sites of nTTCn

TABLE S3. DNA sequences used for GUS reporter assays

Description	Oligo sequence (only upper strand is shown)	
Pi_04701_for_cloning_into_NIFS	5'-GGCCGCGTTCTAGAAGCGTTCTAGAAGCGTTCTAGAAC	Motif is underlined
Pi_20221FL_5s (forward primer)	5'-ACGGGCCCCACAAGAGTAATCATCCAAAG	for amplifying promoter containing PITG_04701 motif; ApaI site underlined
Pi_20221FL_3s (reverse primer)	5'-TCCCCCGGGTGCTCGCCTGTGAGCCAAAA	for amplifying promoter containing PITG_04701 motif; SmaI site underlined
Pi20221_promoter_amplified	5'-<br>ACgggcccCACAAGAGTAATCATCCAAAGTGGTGAGCTGCTACGGCG<br>TAGTACACTGATTCATCGTTCGTCACGGAGGTTATCAAGCAGCGAAA<br>ACCGCACAAAAGCTGTCGAGCAAGAGTTTGCTCTCGTTTGCGCATGT<br>AGCTCGACACATGTAAATCGAAGGTCGTGCTTCACGTTATTAGCGCA<br>AGTTTCTGCCAATGGATTGTGGCTGGTCCAACGCTCAGTGCAGCTTC<br>AACGCCTTGGTCATACCCGAACAGCTCCTCTTTCCGCTCGGATAAAT<br>ATAGCACGCTTCTAGAAACTGCAGCACAACAGTCCATCGTAGCGGCC<br>CATCAGTCACTTCCCAACTAGAAGCGGGCCGCGCAGACCCATCGCCT<br>TTGACGACCAGGAGCGAGCGATCTTGAGTTTCTAGCAGGGAGCGTCA<br>CTCTGCTTCCTGTAGTTTTATTAGCGTCGTTACCAACCGTTTCCAAG<br>TCGCGACAAGAGTGGAGGTTTTGGCTCACAGGCGAGCAcccgggTCC	Product of PCR using the above primers (wild-type promoter); ApaI and XmaI sites used for cloning are in lower case and predicted TF binding sites are underlined.
Pi_20221_mutated	5'-<br>ACgggcccCACAAGAGTAATCATCCAAAGTGGTGAGCTGCTACGGCG<br>TAGTACACTGATTCATCGTTCGTCACGGAGGTTATCAAGCAGCGAAA<br>ACCGCACAAAAGCTGTCGAGCAAGAGTTTGCTCTCGTTTGCGCATGT<br>AGCTCGACACATGTAAATCGAAGGTCGTGCTTCACGTTATTAGCGCA<br>AGTTTCTGCCAATGGATTGTGGCTGGTCCAACGCTCAGTGCAGCTTC<br>AACGCCTTGGTCATACCCGAACAGCTCCTCTTCCGCTCTGATAAATA<br>TAGCACGCGCAACTCCCAGTAAGCACAACAGTCCATCGTAGCGGCCC<br>CTCAGTCACGCAACTCCCAGTAGCGGGCCGCGCAGACCCATCGCCTT<br>TGACGACCAGGAGCGAGCGATCTTGAGTTTCTAGCAGGGAGCGTCAC<br>TCTGCTTCCTGTAGTTTTATTAGCGTCGTTACCAACTGTTTCCAAGT<br>CGCGACAAGAGTGGAGGTTTTGGCTCACAGGCGAGCAcccgggTCC	Synthesized version of the above with the TF binding site mutated.
